# A side-effect-free chemotactic-antibacterial wound dressing for programmatic trapping, killing of bacteria, and wound repair

**DOI:** 10.1016/j.mtbio.2025.102594

**Published:** 2025-11-25

**Authors:** Zhiyi Liao, Xinxin Su, Tingna Luo, Xiaohong Zhao, Yicheng Guo, Xisheng Xu, Fan Wang, Gaoxing Luo, Rixing Zhan

**Affiliations:** a Institute of Burn Research, State Key Laboratory of Trauma and Chemical Poisoning, Southwest Hospital, The Third Military Medical University (Army Medical University), Chongqing, 400038, China; b Department of Burn and Plastic Surgery, the First People's Hospital of Chenzhou, University of South China, Chenzhou, 423000, China; c Department of Burn, Plastic and Aesthetic Surgery, the first Affiliated Hospital of Guilin Medical University, Guilin, 541001, Guangxi, China; d Department of Plastic and Reconstructive Surgery, Southwest Hospital, The Third Military Medical University (Army Medical University), Chongqing, 400038, China

**Keywords:** Wound infection, Chemotactic antibacterial strategy, RGD-Functionalized hydrogel, Controlled ion release, Tissue regeneration

## Abstract

Wound infection is a common clinical complication in surgery and war trauma, which can trigger an adverse pathological state of local and systemic inflammatory response. Although antibiotics were once regarded as an important means to suppress wound infections, their frequent use can lead to bacterial resistance and delay tissue healing and regeneration. In order to solve these problems, based on previous research, we screened and designed a chemotactic, antibacterial dressing that promotes wound healing. First, an arginine-glycine-aspartate tripeptide (RGD) with a significant chemotactic effect on *Escherichia coli* was systematically screened through chemotactic experiments in vitro. The RGD was grafted to gelatin methacrylated (GelMA), which was then combined with carboxymethyl chitosan (CMCS)-wrapped silver nanoparticles to form a composite hydrogel. Finally, transplanting the composite hydrogel into infected wound models in mice and rabbits can directionally attract bacteria, maintain the long-term bactericidal ability of silver ions, and promote wound healing. Pathway enrichment analysis showed that RGD significantly improved the mobility of *Escherichia coli* by activating the chemotaxis pathways. The hydrogel transplanted onto the wound surface further inhibits inflammatory signaling pathways such as NF-κB through antibacterial activity, thereby providing a basis for wound regeneration (significant activation of Wnt and ECM pathways). These results indicate that the chemotactic-antibacterial wound dressing provides a novel, side-effect-free treatment method, that provides an optimal wound microenvironment for promoting wound healing. This provides a new strategy for the treatment of clinically infected wounds.

## Graphical abstract

**Figure:**
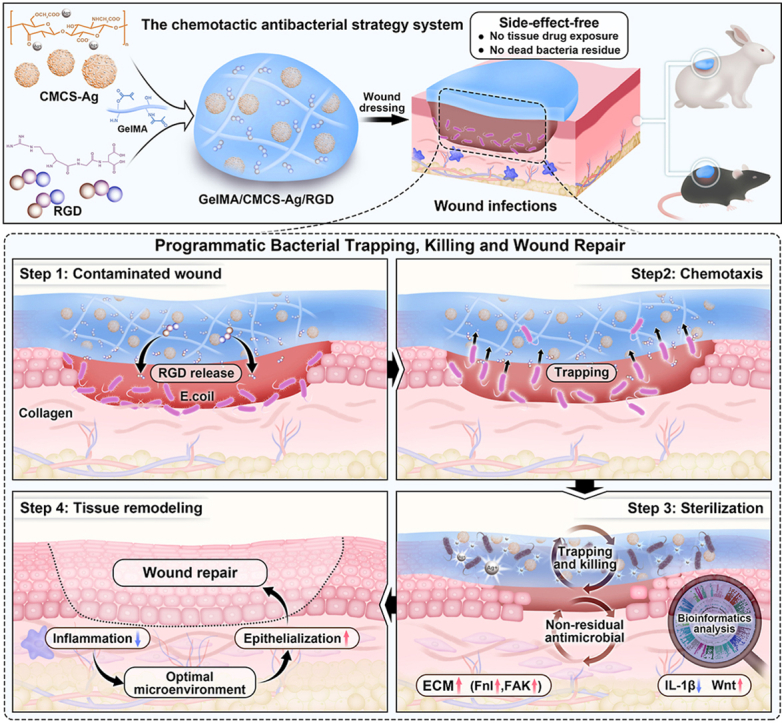

## 1 Introduction

Wound infection, which remains a critical clinical challenge in surgery and war trauma, can trigger tissue inflammation and necrosis, delay wound healing, and even lead to life-threatening systemic infections [1,2]. Although conventional antibiotic therapy is widely used, it has become increasingly ineffective due to the escalating issue of antibiotic resistance [3,4]. While emerging strategies such as targeted antimicrobials [5], phage therapy [6,7], and resistance mechanism-targeted bacterial vaccines [8,9] show promise, their long development cycles and unvalidated safety/efficacy profiles hinder their ability to effectively tackle the growing crisis of bacterial drug resistance in the short term. Therefore, the urgent development of an innovative anti-infective strategy is indispensable [10].

Current antimicrobial therapeutic strategies inevitably leave bacterial and drug residues [11,12], trigger substantial tissue inflammation due to the massive release of bacterial lysates after sterilization, and impede wound healing via residual drug toxicity. For instance, silver sulfadiazine, commonly used for burns, frequently leaves harmful metal residuesand exerts toxic effects [13]. Bacterial chemotaxis, a biological phenomenon where bacteria detect chemical gradients through specialized receptors and adjust their movement patterns—moving toward beneficial stimuli or away from harmful ones via intricate signaling networks—presents a promising avenue for developing innovative strategies to eliminate bacterial populations from infected tissues [14,15]. We previously exploited bacterial chemotaxis to design a novel antimicrobial system (L-Lys/HHC-36-PDA@GO) targeting *Pseudomonas aeruginosa* [16]. This system effectively extracts bacteria from infected tissues, enables programmatic trapping and killing (In this study, we uniformly use the term ‘programmatic trapping and killing’ to describe the mechanism by which the hydrogel exerts synergistic antibacterial effects through chemotactic attraction and silver ions), and minimizes collateral tissue damage. However, whether this chemotactic bactericidal strategy can be extended to other common wound-infecting pathogens remains unclear and requires further exploration.

Infection management alone is insufficient because bioactive materials must cooperatively support wound healing [17]. Hydrogels are well established as therapeutic agents in wound repair due to their structurally flexible and biologically responsive natures [[18], [19], [20]]. One such excellent example is GelMA (Gelatin Methacryloyl), a gelatin-based hydrogel, which is well acknowledged for structural flexibility and biological responsiveness [20]. Our previous studies have established that GelMA hydrogels can facilitate tissue regeneration in traumatic hemostasis and tissue injury repair [21]. GelMA retains the RGD motif [22], which binds to αvβ3 and α5β1 integrins on the cell surface, inducing integrin clustering and subsequent phosphorylation of focal adhesion kinase [23,24]. This activates downstream cascades, including the Src-dependent PI3K-AKT and MAPK pathways, as well as RhoGTPase-mediated cytoskeletal reorganization, collectively regulating cell adhesion, migration, and survival [25]. CMCS, a water-soluble derivative of chitosan, possesses excellent biocompatibility and film-forming properties. When used to encapsulate silver nanoparticles (AgNPs), CMCS can stabilize the particles and enable sustained Ag^+^ release, thereby reducing cytotoxicity and prolonging antibacterial activity. These unique properties make CMCS-Ag a promising component for constructing multifunctional antibacterial hydrogels and wound dressings.

**Scheme 1:**
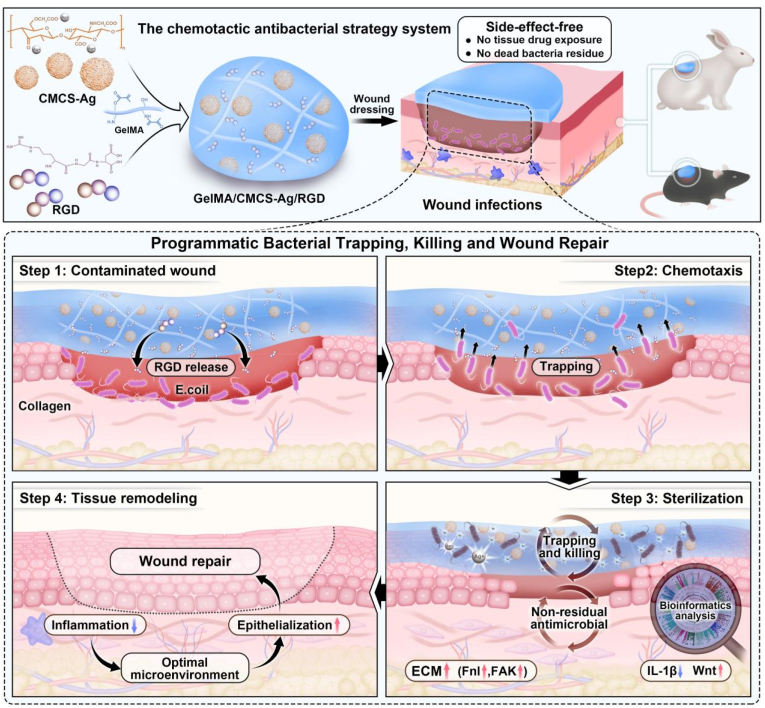
Schematic illustration of the chemotactic antimicrobial strategy.

This research improved upon the previous study and employed the chemotactic bactericidal process in *E. coli* and other wound-pathogenic bacteria [16], which are prevalent in infected wounds. Bioactivities for tissue restoration were enhanced, and bacterial chemotaxis was harnessed, via the use of the cell-adhesive peptide RGD (Scheme 1). In vivo and in vitro studies, which were highly extensive, validated the role of GelMA/CMCS-Ag/RGD hydrogel in entrapping and killing bacteria, and accelerating the wound-healing process, including helping ECM remodeling, inhibiting inflammation through Fn1, FAK, IL1β and Wnt pathways. The effectiveness was also validated while being tested on wounds in rabbits, which indicates it has huge potential to be employed by humans in the future.

## 2 Materials and methods

### 2.1 Materials

Silver nitrate solution (0.1 mol/L, 0.1 N; AgNO_3_, MW 169.87) and CMCS (degree of substitution ≥90 %) were obtained from Aladdin Reagent Co. (Shanghai, China). (S)-2-(2-((S)-2-Amino-5-guanidinopentanamido)-acetamido) succinic acid (RGD, MW 346.34, purity >98 %) was purchased from Bidepharm Technology Co. (Shanghai, China). Porcine gelatin (type A, powder, from porcine skin, gel strength ∼300 g Bloom) and methacrylic anhydride (≥98 %, containing 2000 ppm topanol A as inhibitor, MW 154.16) were supplied by Sigma-Aldrich (Shanghai, China). Lithium phenyl (2,4,6-trimethylbenzoyl) phosphinate (EFL-LAP, MW 296.23, purity >99.8 %) was provided by Engineering for Life Intelligent Equipment Co. (Suzhou, China). Lysine (purity >99 %), arginine, glycine, serine, and aspartic acid were purchased from Aladdin Reagent Co. (Shanghai, China). Prepared plate media was obtained from Hope Bio-Technology Co. (Qingdao, China). Collagenase type I (360 U/mg DW) was supplied by Diamond Biotechnology Co. Calcein/PI Cell Viability/Cytotoxicity Assay Kit, Antifade Mounting Medium with DAPI, and phosphate-buffered saline (PBS, 1X, premixed powder) were provided by Beyotime Biotechnology Co. Ultrapure water was provided by Heal Force water purification systems. The Cell Bank of Typical Culture Collection, Chinese Academy of Sciences (China) supplied NIH 3T3 fibroblasts. The *Escherichia coli* (*E. coli*), *Pseudomonas aeruginosa (PA)* and *Staphylococcus aureus (SA)* strains were provided by the microbiological laboratory, Department of Burns, Southwest Hospital, First Affiliated Hospital of Army Medical University (Chongqing, China). The chemotaxis-deficient mutant (*CheA-type E. coli*) was provided by Hangzhou Baosai Biotechnology Co., Ltd. Mepilex antimicrobial soft silicone foam dressing and Atrauman Ag dressing were provided by the First Affiliated Hospital of Army Medical University. Male BALB/c mice (6–8 weeks, 20–25 g), SD rats (6–8 weeks, approximately 200 g), and New Zealand white rabbits (6–8 weeks, approximately 1000 g) were provided by the Third Military Medical University, Laboratorial Animal Department. All animal experimentation was conducted according to protocols approved by the First Affiliated Hospital of Army Medical University's Ethics Committee, with the approval number AMUWEC20224129.

### 2.2 Material synthesis methods

#### 2.2.1 Synthesis of CMCS-Ag

First, dissolve 2 g of CMCS powder in 49 mL of deionized water and heat to 80 °C until completely dissolved. Allow the temperature to drop below 50 °C, then slowly add 1 mL of silver nitrate solution in the dark while stirring magnetically. After mixing well, the CMCS-Ag complex formed upon exposure to UV light for 30 min.

#### 2.2.2 Synthesis of GelMA

Add 10 g of gelatin to 100 mL of PBS, heat to 60 °C, and stir magnetically for 2 h until dissolved. Slowly add 9 mL of methacrylic anhydride and react at 55 °C for 3 h until the solution turns from transparent to milky white. Then add 400 mL of PBS (1:4 ratio) to terminate the reaction. Dialyze the mixture with deionized water for 7 d, changing the water every 8 h. Centrifuge the mixture at 4500 rpm for 5 min, store at −80 °C for 4–5 h, and then freeze-dry for 4–5 days.

#### 2.2.3 Synthesis of GelMA/CMCS-Ag/RGD hydrogel

GelMA (1 g, 10 % w/v) was dissolved in CMCS-Ag solution (10 mL), and RGD (0.2 g, 2 w/v%) and LAP (0.05 g, 0.5 w/v%) were added. The mixture was magnetically stirred until he components had completely dissolved to produce the hydrogel precursor solution, which was irradiated under UV light for 5 min to generate the hydrogel.

### 2.3 Structure characterization

Purified CMCS-Ag was examined using scanning electron microscopy (SEM) and transmission electron microscopy (TEM). Size and dispersion were investigated using nanoparticle size measurement techniques. The absorbance spectrum of the antimicrobial CMCS-Ag composite particles was acquired using UV–vis spectrophotometry in the wavelength range of 200–800 nm.Hydrogel samples were collected, preserved overnight at −80 °C, and freeze-dried at −60 °C for 48 h. The dried samples were mounted on a sample stage using conductive adhesive and sputter-coated with gold for 60 s. The internal structure was characterized by SEM. Fourier-transform infrared spectroscopy (FTIR) spectroscopy was performed using dried hydrogel samples.

### 2.4 Rheological characterization

Hydrogel rheological properties were measured using a TA DHR-2 rheometer. We tested the oscillatory strain dependence of storage modulus (G′) and loss modulus (G″) were examined at a fixed strain of 0.5 % and a frequency range of 0.1–100 rad/s, respectively. All the measurements were conducted at room temperature.

### 2.5 Swelling tests

Hydrogel discs (8 mm in diameter) were incubated in deionized water at 37 °C under 5 % CO_2_ for 2 h. After blotting the surface with filter paper, we recorded the mass of the empty Eppendorf tube (Ms) and the combined mass of the tube plus the hydrated hydrogels (M_A_). The samples were then lyophilized for 48 h and reweighed (M_B_). The swelling ratio was calculated as follows:

$$\text{Mass}\;\text{swelling}\;\text{rate}\;\left(\%\right)=\left[\left(M_{A}-M_{S}\right)-\left(M_{B}-M_{S}\right)\right]/\left(M_{B}-M_{S}\right)\times 100\%\text{.}$$

### 2.6 Water retention capacity

After hydrating for 2 h in deionized water, the surface of the hydrogels was blotted, and the initial mass was recorded (M_0_). Samples in open 1.5 mL Eppendorf tubes were maintained at ambient temperature, and we recorded the residual mass (M_1_) at 0, 1.5, 3, 6, 12, 24 and 48 h. Water retention was calculated as follows:

$$\text{Water}\;\text{retention}\;\text{rate}\;\left(\%\right)=\left(M_{1}-M_{0}\right)/M_{0}\times 100\%$$

### 2.7 In vitro degradation

Eppendorf tubes were weighed (M_0_)and record the initial mass of 8-mm hydrogel discs as (M1).Load the hydrogel into the Eppendorf tubes. The samples were immersed in 1 mL collagenase type I solution (2 U/mL) and incubated at 37 °C under 5 % CO_2_ with daily 1 mL enzyme replacement. At designated time points (0, 3, 5 and 7 d),the hydrogels were lyophilized n the tubes and weighed again (M_2_). The mass retention rate was calculated as: Mass remaining rate (%) = [(M_1_ - M_0_) - (M_2_ - M_0_)]/(M_1_ - M_0_) × 100 %

### 2.8 Mechanical property tests

Cylindrical hydrogel samples (1 cm in diameter, 3 mm in thickness) were prepared. and the compressive properties were measured using a universal testing machine (CMT6103, China Instrument Industrial Systems Co., Ltd.) with a crosshead speed of 10 mm/min, following standard GB/T 1041–2008. The compressive strength (P) at rupture was calculated by dividing the applied force by the cross-sectional area of the sample. Each group included at least three parallel samples. After compressing the hydrogel by 80 %, 5 %–15 % of the compression curve is taken for linear fitting, and the slope is defined as the compression modulus of the hydrogel.

### 2.9 Silver ion (Ag^+^) release curve analysis

To evaluate the in vitro release of Ag^+^, the GelMA/CMCS-Ag/RGD hydrogel (1 mL) was immersed in 10 mL of sterile 0.9 % NaCl solution, and the reaction vessel was sealed and incubated at 37 °C with agitation (100 rpm). For 7 consecutive days, 1 mL of solution was withdrawn and replaced with fresh NaCl solution. The Ag^+^ concentration was quantified using inductively coupled plasma-mass spectrometry (ICP-MS) after 100-fold dilution of the sample, and these data were used to plot a cumulative release curve. For further analysis of Ag^+^ retention and release, the hydrogel (1 mL) was immersed in deionized water for 3 and 7 d, lyophilized, and cross-sections of the samples were analyzed by scanning electron microscopy-energy-dispersive X-ray spectroscopy (SEM-EDS).The elemental distribution and relative silver content were recorded.

Additionally, to better simulate the physiological degradation environment, a collagenase-containing release assay was performed. The GelMA/CMCS-Ag/RGD hydrogel (1 mL) was immersed in 10 mL of 0.9 % NaCl solution containing Type I collagenase (2 U/mL) and incubated at 37 °C with shaking (100 rpm). Each day for 3 consecutive days, 1 mL of the supernatant was withdrawn and replaced with fresh collagenase solution. The Ag^+^ concentration was determined by ICP-MS after 100-fold dilution, and a cumulative release curve was plotted.

### 2.10 RGD release curve analysis

To measure RGD peptide release from GelMA/CMCS-Ag/RGD hydrogels, a bicinchoninic acid (BCA) protein assay-based standard curve was constructedby plotting the concentration (ranging from 0 to 1000 μg/mL) as a function of the absorbance at 562 nm. For the assay, 200 μL BCA reagent and 25 μL RGD-containing supernatant were combined. Hydrogel discs, with a diameter of 8 mm were placed in 1 mL of saline and incubated in a humid atmosphere of 37 °C under 5 % CO_2_. At the designated times (1.5, 3, 6, 12, 24, and 48 h), the supernatants were collected after centrifugation, and the RGD concentration was measured using the BCA assay.

Similarly, the release behavior of RGD in an enzymatic environment was assessed by immersing the hydrogel discs in 1 mL of 0.9 % NaCl solution containing Type I collagenase (2 U/mL) at 37 °C under 5 % CO_2_. Supernatants were collected at 1.5, 3, 6, 12, 24, 48, and 72 h and analyzed using the BCA method. The cumulative release curve was plotted to evaluate RGD stability under enzymatic degradation.

### 2.11 Biocompatibility evaluation

#### 2.11.1 Cytotoxicity in vitro

200 μL of sterilized hydrogel samples were added to 500 μL of Dulbecco's modified eagle medium (DMEM), supplemented with 10 % fetal bovine serum (FBS) and 2 % penicillin-streptomycin,and incubated at 37 °C for 24 h, after which the leachate was collected and filtered through sterile 0.22 μm filters. NIH 3T3 fibroblasts (2000 cells per well) were seeded into 96-well plates and incubated at 37 °C for 24 h. Next, 100 μL of the leachate (experimental group) or DMEM (control group) was added, and the cells were incubated at 37 °C for 24, 48, and 72 h. The medium was removed after incubation, and 100 μL of DMEM with 10 % CCK-8 solution was added to each well. The mixture was incubation for 2 h at 37 °C. A microplate reader (Thermo Scientific, USA) was used to measure the absorbance at 450 nm. and OD values were used to determine cell viability.

#### 2.11.2 Cell fluorescence staining

A Beyotime Biotechnology Co. live-dead assay kit) was used according to the manufacturer's instructions to determine cell viability after incubation for 24, 48, and 72 h, as described above. The cells were washed three times with PBS and stained with propidium iodide (PI) and calcein AM for 30 min to differentiate dead and living cells. The cells were counterstained with DAPI, and the fluorescence intensity was analyzed by laser confocal microscopy. Images were captured and processed using ImageJ software.

#### 2.11.3 Hemolysis assay

Blood samples from BALB/c mice(20 g) were collected in heparin-containing tubes. The collected red blood cells (RBCs) were diluted in saline, washed, and centrifuged (1500 rpm, 5 min) three times. Red blood cell dispersions composed of 200 μL of RBCs in 4 mL saline were prepared,and 100 μL RBC dispersion and 900 μL of a mixture of saline and hydrogel sample were mixed. Ultrapure water (900 μL) served as the positive control, and saline was utilized as the negative control. The samples were incubated at 37 °C for 3 h and then centrifuged (1500 rpm, 15 min), after which the absorbance was measured at 540 nm. The hemolysis rate was calculated as follows: Hemolysis rate (%) = (A - C)/(B - C) × 100 %, where C is the negative control absorbance, and B is the positive control absorbance.

#### 2.11.4 Subcutaneous implantation

Hydrogels (8 mm in diameter × 3 mm in height) were embedded under the skin of male rats (200 g, n = 4) to evaluate its biocompatibility in vivo. Seven days later, the rats were sacrificed. Subcutaneous tissues and major organs (heart, liver, spleen, lung, and kidney) of the rats were taken and fixed for histological analysis of hematoxylin and eosin (H&E).

### 2.12 In vitro bacterial chemotaxis assay

#### 2.12.1 Bacterial culture

*E. coli* (ATCC 25922) was cultured in LB broth at 37 °C with shaking at 220 rpm for 12 h. During exponential growth, *E. coli* bacteria were collected by centrifugation (4500 rpm, 5 min), and resuspended in PBS.

#### 2.12.2 Chemotaxis assay of chemoattractant

To evaluate the chemotactic response of *E. coli*, two complementary assays were performed: a radial well-based directional migration assay and a concentric ring-based agar diffusion assay. Filtered solutions (10 mM) of lysine, arginine, glycine, serine, aspartic acid, and sterilized PBS were prepared for these experiments.

Radial well-based directional chemotaxis assay: Motility agar was prepared by dissolving 0.9 g of yeast extract, 3 g of peptone, 1.5 g of NaCl, and 2.4 g of agar powder in 300 mL of ultrapure water. The solution was boiled, autoclaved (121 °C for 20 min), and poured into 100 mm Petri dishes (15 mL per plate). After solidification, wells (one central well and 6–8 peripheral wells) were punched into the agar using sterile pipette tips. Bacterial suspension (100 μL) was added to the central well. And the prepared amino acid chemotaxis solutions and PBS (50 μL each) were added to peripheral wells. The dishes were allowed to stand for 30 min for initial diffusion and then incubated at 37 °C under 5 % CO_2_. Photographs were taken at 12, 16, and 20 h and distances of bacterial migration in the central wells and colony growth in peripheral wells were analyzed using an automated colony counter. The chemotactic effects of RGD (1 %, 2 %, and 4 %) and the influence of different distances (2.5 mm, 5 mm, and 10 mm) from the central well similarly tested, with sterile water as the control and 2 % RGD as the experimental group.

Concentric ring assay: Concentric agar plates were prepared by mixing 0.3 g of yeast, 1 g of peptone, 0.25 g of agar powder, 0.5 g of NaCl, and 100 mL of deionized water in an triangular flask, sealing the mouth with sealing film, and autoclaving for 20 min. The agar solution appeared light yellow, clear, and transparent. 15 mL of agar solution was added to each 100 mm culture dishes as the control. 3 mL of amino acid chemotaxis solution and 12 mL of agar solution were added as a mixture of the experimental groups. The plates were then cooled at RT for 4 h and stored at 4 °C refrigerator. A dried, autoclaved filter paper disc (6 mm in diameter) was placed at the center of each agar plate. Add 5 μL of bacterial suspension to completely wet the filter paper. The plates were incubated at 37 °C under 5 % CO_2_. The concentric agar plates we made do not hinder the movement of bacteria, which will spread from the central filter paper to the periphery. Images were acquired at 12, 16, and 20 h, and the colony areas were measured using a colony counter. Additional assays were similarly conducted with 5 %, 10 %, and 20 % UV-irradiated GelMA solutions and 0.5 %, 1 %, 2 %, and 4 % bacteria-filtered RGD solutions. PBS and pure agar served as controls.

Based on the results of these two assays, the chemotaxis ability of bacteria is quantitatively evaluated. All data were independently analyzed by two researchers under blinded conditions to ensure the consistency and objectivity of the results.

#### 2.12.3 Chemotactic specificity and pathway validation of RGD toward bacteria

To further investigate the chemotactic specificity and signaling mechanism of RGD, two additional assays were performed using the same radial well-based directional migration setup described above. Firstly, we evaluated the chemotactic effects of 2 % RGD on *PA* (Gram-negative) and *SA* (Gram-positive). Add the bacterial suspension (100 μL,OD_600_ = 0.6) to the central well. Place 2 % RGD solution (50 μL) in half of the surrounding Wells at intervals of 2.5 mm, and sterile water in the other half. The plates were incubated at 37 °C under 5 % CO_2_ for 12 h, and bacterial migration was recorded by photography. The migration distance and aggregation area were quantified using ImageJ software. To further verify the signaling pathways of RGD chemotactic bacteria, assays were conducted on *E. coli* and a chemotaxis-deficient mutant (*CheA-type E. coli*) under the same conditions. As mentioned above, the directional migration behavior was observed and analyzed. Each analysis was conducted three times, and the results were expressed as mean ± standard deviation (SD).

#### 2.12.4 Evaluation of chemotactic effects of hydrogels

Hydrogels were prepared in confocal Petri dishes, and half of the hydrogel was removed with a sterile blade. The remaining portion was fully hydrated with sterilized deionized water for 2 h, after which excess water was aspirated. An *E. coli* suspension (OD_600_ = 0.6, expressing GFP via plasmid) was applied. Bacterial movement was immediately recorded by laser confocal microscopy.

#### 2.12.5 Chemotaxis transcriptomic analysis

To investigate the chemotactic mechanism of GelMA/CMCS-Ag/RGD on *E. coli*, total RNA was extracted and subjected to transcriptome sequencing. Library preparation and sequencing were performed using an Illumina NovaSeq™ X Plus platform. Bioinformatic analyses, including read mapping, expression quantification, differential expression, and KEGG pathway enrichment, were conducted via the Majorbio Cloud Platform using standard pipelines and tools (Bowtie2, RSEM, DESeq2, and KOBAS).

### 2.13 In vitro antibacterial assay

*E. coli* in the logarithmic growth phase was prepared as a suspension at an OD_600_ of 0.6 in LB broth. A 100 μL aliquot of bacterial suspension was evenly spread on plate-counting agar. We placed the hydrogel samples on the surface of the agar and allowed the bacteria to grow at 37 °C. Photographs were taken of the inhibition circle that had been incubated. Meanwhile, we further explored the antibacterial effect of the hydrogel on various bacteria through the inhibition circle assay and compared it with two commercial dressings used in clinical practice. Additionally, 1 mL of bacterial suspension was added to each well of a 48-well plate containing hydrogel samples from each group. The plates were shaken at 37 °C and 100 rpm for 24 h. Then, 200 μL of each sample were removed to measure the optical density at 600 nm (OD_600_). Moreover, we diluted 100 μL of bacterial suspension 10^6^-fold in PBS, poured the mixture evenly onto a plate with nutrient agar, and then counted the number of colony-forming units (CFUs) after 12 h of incubation at 37 °C.

### 2.14 In vivo animal studies

#### 2.14.1 Wound healing assay in BALB/c mice

Full-thickness skin wound was established in 36 male BALB/c mice anesthetized via intraperitoneal injection of pentobarbital. The mice were randomly divided into six groups: the blank (uninfected), GelMA/CMCS-Ag/RGD, Drug-resistant, GelMA/CMCS-Ag, and GelMA groups. After hair removal, two full-thickness wounds (8 mm in diameter) were created on the dorsal skin of each mouse. The wounds were inoculated with 5 μL of *E. coli* suspension (1 × 10^8^ CFU/mL); the resistant group received an antibiotic-resistant *E. coli strain*. The hydrogels were applied according to group assignment, and wounds were covered with sterile gauze. The dressings were changed and the wounds were photographed after 1, 3, 5, and 7 d. On days 1, 3, and 7, bacterial samples were collected from the wound secretions using sterile swabs, resuspended in 8 mL of saline, and quantified by plate counting. Additionally, the wound tissues were excised, homogenized in saline, and similarly enumerated. On days 3 and 7, the wound tissues were fixed in 4 % paraformaldehyde, embedded in paraffin, sectioned, and stained with H&E and Masson's trichrome. The lengths of the new epithelial tissue were measured by two independent observers using ImageJ. After 7 d, the major organs (liver, heart, spleen, kidney, and lung) were collected for histological examination ia H&E staining. All the experiments were performed in triplicate.

#### 2.14.2 Transcriptomic analysis of RGD in wound healing

To elucidate the mechanism underlying GelMA/CMCS-Ag/RGD-mediated wound healing, wound tissues were harvested 3 and 7 d post-treatment, (n = 3 per time point) for transcriptome analysis. Total RNA was extracted, sequenced, and analyzed as described in section 2.12.4.

#### 2.14.3 Wound healing experiments in rabbits

Six New Zealand rabbits were randomly divided into two groups (experimental and control, n = 3 per group). Animal experiments were performed with n = 3 per group. This sample size was determined based on preliminary observations of a strong effect size, which was sufficient to achieve statistical power for detecting intergroup differences. Three full-thickness wounds (2.5 × 2.5 cm^2^) were created on the dorsal.surface of each rabbit and inoculated with 20 μL of *E. coli* suspension, which was evenly spread using sterile swabs. The experimental wounds were covered with the hydrogel, whereas the control wounds remained untreated but covered with gauze. The dressings were changed daily, and the wound scabs were trimmed every five days. Wounds were photographed 5, 11, and 21 d later, after which the tissues were excised and subjected to histological analysis via staining with Masson's and H&E.

### 2.15 Data analysis

The experimental data were analyzed using Origin 2021 and Adobe Illustrator 2023 software. The results are expressed as the means ± standard deviation (SDs). Statistical significance between groups was evaluated by one-way ANOVA or two-way ANOVA. Differences were considered statistically significant at *∗P* < 0.05, and highly significant at *∗∗P* < 0.01, *∗∗∗P* < 0.001, and nonsignificant differences were denoted as ns.

## 3 Results and discussion

### 3.1 Screening of chemotactic agents for *E. coli* and their chemotactic mechanisms

**Fig. 1:**
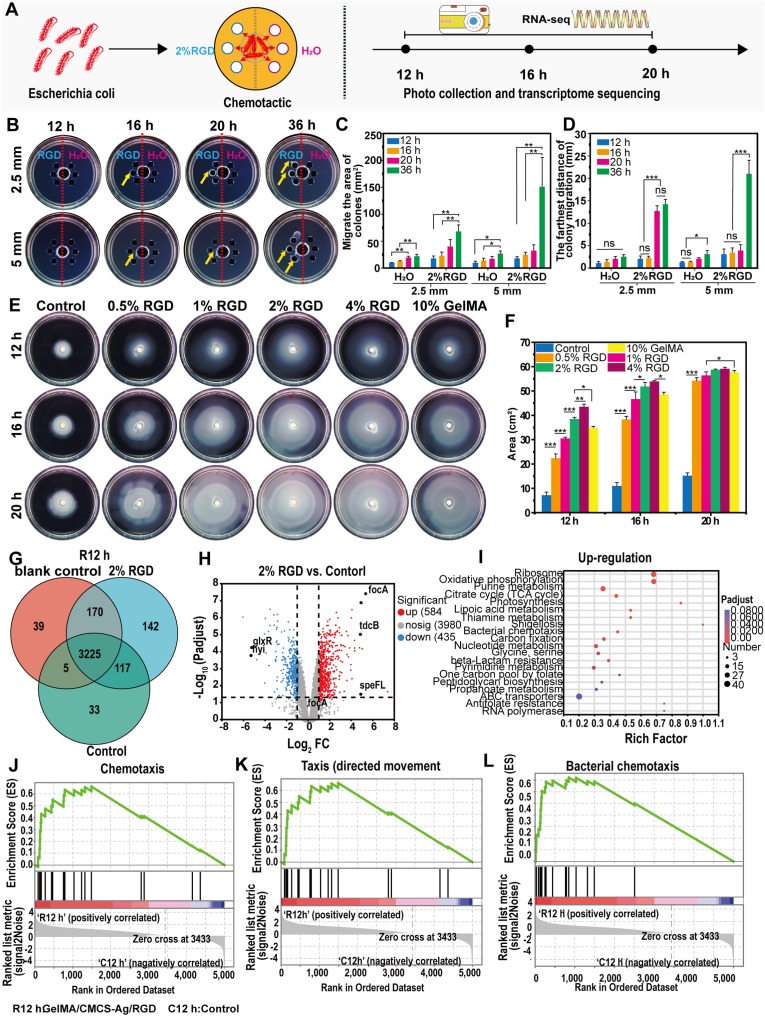
In vitro bacterial chemotaxis screening and chemotaxis mechanism. (A) Schematic diagram of in vitro bacterial chemotaxis screening and research on the chemotaxis mechanism. (B) Chemotactic effect of RGD peptide on *E. coli* at different distances. (C) Statistical result of chemotaxis area. (D) Statistical result of chemotaxis distance (n = 3). (E) Results of concentric ring assay of *E. coli* at different RGD concentrations. (F) Statistical result of concentric ring assay's area (n = 3). (G) Venn diagram analysis results of overlapping DEGs among the 2 % RGD group, control group, and blank group. (H) Volcano plot enrichment results for genes upregulated and downregulated after 2 % RGD treatment. (I) KEGG enrichment analysis of upregulated DEGs after 2 % RGD treatment. (J–L) GSEA plots showing significant enrichment of chemotaxis-related gene sets after 2 % RGD treatment. (*∗P* < 0.05, *∗∗P* < 0.01, *∗∗∗P* < 0.001, ns: non-significant differences).

Our previous study demonstrated that L-lysine exhibited chemotactic activity toward *Pseudomonas aeruginosa*, but had a relatively weak effect on *E. coli* [16]. The literature indicates that serine, asparagine, and aspartic acid attract *E. coli*, though their specific mechanisms have remain unclear [26]. Interestingly, aspartic acid, arginine, and glycine form the RGD peptide sequence, a bioactive motif well-known for cell adhesion and tissue regeneration but less studied in bacterial chemotaxis, and we speculate that the chemotactic effect of RGD in *E. coli* may be stronger [27]. To systematically screen amino acids for their chemotactic effects on *E. coli*, we selected lysine, arginine, glycine, serine, and aspartic acid (Fig. 1A). It was found that these amino acids induced different degrees of chemotactic migration in *E. coli*, with RGD displaying the strongest effect (Fig. S1A). This result is consistent with previous reports [26].

Further investigation of the potential of different distances (2.5, 5, and 10 mm) and RGD concentrations (1 %, 2 %, and 4 %) on *E. coli* chemotaxis revealed that 2 % RGD exhibited significant chemotactic activity at a distance of 2.5 mm (Fig. 1B–D and Fig. S1B–1C). Since GelMA contains RGD sequences, we hypothesized that RGD may promote bacterial proliferation. The results showed that the bacterial diffusion areas increased with increasing RGD concentration, evidencing a dose-dependent chemotactic and growth response (Fig. 1E–F and S1D–1E). However, maximum GelMA diffusion occurred at a concentration of 10 %, after which diffusion decreased, perhaps due to the increased density of the hydrogel, which impeded bacterial mobility. Notably, appreciable chemotactic activity was observed at early time points (12 h), indicating that RGD is an effective, time-dependent chemoattractant for *E. coli* chemoattractant.

To further evaluate the chemotactic specificity of RGD, we tested its effect on other bacterial species and mutants. When the distance between the hydrogel and bacterial inoculation site was 2.5 mm and the RGD concentration was 2 %, a pronounced chemotactic response was observed in *PA* (Gram-negative), whereas *SA* (Gram-positive) exhibited negligible migration (Supplementary Fig. S1E–1G). These findings indicate that the chemotactic effect of RGD is specific to Gram-negative bacteria, likely related to their flagella-driven motility and receptor-mediated sensing. This selective chemotaxis further supports the rational design of RGD-based “programmatic trapping” antibacterial dressings. In addition, to confirm the signaling pathway involved in RGD-induced chemotaxis, we compared the responses of *E. coli* and a chemotaxis-deficient mutant (*CheA-*). As shown in Supplementary Fig. S1E–1G, the *E. coli* showed a clear directional migration toward the RGD-modified hydrogel, whereas the *CheA-*mutant exhibited almost no movement. This finding demonstrates that RGD-induced chemotaxis is mediated through the *CheA*-dependent signaling cascade, confirming a true chemotactic activation rather than passive diffusion. Following these results, we directed further studies toward the biological pathways mediating RGD's effects. The evaluation of bacterial induction ability did not rely on direct visual inspection of images but was quantitatively determined by measuring the migration distance and bacterial aggregation density in chemotaxis assays. To ensure objectivity, the raw data were anonymized and independently analyzed by two researchers under blinded conditions.

To better understand the signaling pathways that RGD uses to induce chemotaxis, we first identified key differentially expressed genes (DEGs) through transcriptomic analysis. Correlation heatmap analysis showed that the higher the similarity between samples within a group, the better the repeatability (Fig. S2A). The Venn diagram analysis showed that there were 3225 DEGs common to the blank control, control, and 2 % RGD groups at 12 h, and 3218 DEGs that were the same among all the groups (Fig. 1G–S2B). At 12 h, there were 1274 DEGs when comparing the 2 % RGD group to the blank control and 1105 DEGs comparing the 2 % RGD group to the control (Fig. S2C and 1E). This indicated that RGD significantly activated the transcription of *E. coli.* It demonstrated that the 2 % RGD group had 584 upregulated and 435 downregulated genes compared to the control group (Fig. 1H). Previous research showed that aspartic acid strongly attracts *E. coli*. We hypothesized that RGD activated pathways related to chemotaxis because it contains aspartic acid [16]. The CheA gene encodes autophosphorylating histidine kinase, which phosphorylates downstream targets *CheY* and *CheB*. *CheA*, along with transmembrane chemoreceptors and *CheW*, transduces signals from the environment. This supports the hypothesis that RGD increases the bacteria's mobility and ability to detect direction [28]. Observation showed that treatment with 2 % RGD had several chemotaxis-associated gene activations (*CheA, CheB, CheW,* and *CheY*) compared to controls (Table 1).

**Table 1:** Differential expression of core chemotaxis genes in E. coli. Normalized expression values (see Methods) for the canonical chemotaxis module-cheA (histidine kinase, PQQ28_RS10570), cheB (methylesterase, PQQ28_RS10595), cheW (adaptor, PQQ28_RS10575), and cheY (response regulator, PQQ28_RS10600)-are shown for five biological replicates per condition at 12 h and 16 h. Columns “R12h_1-5” and “R16h_1-5” denote the treatment group; “C12h_1-5” and “C16h_1-5” denote matched controls.

**Gene ID**	**Gene Name**	**R12h_1**	**R12h_2**	**R12h_3**	**R12h_4**	**R12h_5**	**C12h_1**	**C12h_2**	**C12h_3**	**C12h_4**	**C12h_5**
PQQ28_RS10570	cheA	74.42	64.58	52.31	42.22	67.93	25.98	14.02	15.88	13.89	17.25
PQQ28_RS10595	cheB	29.65	27.45	26.27	22.48	31.14	14.57	11.19	9.01	7.76	11.6
PQQ28_RS10575	cheW	159.11	164.76	127.81	108.84	187.93	66.75	56.14	54.46	61.63	72.54
PQQ28_RS10600	cheY	97.8	92.38	64.7	57.87	61.63	28.79	19.96	31.91	31.28	26.76

**Gene ID**	**Gene Name**	**R16h_1**	**R16h_2**	**R16h_3**	**R16h_4**	**R16h_5**	**C16h_1**	**C16h_2**	**C16h_3**	**C16h_4**	**C16h_5**

PQQ28_RS10570	cheA	64.8	45.22	87.61	54.88	86.65	50.56	47.71	66.5	57.57	69.13
PQQ28_RS10595	cheB	32.94	22.29	63.38	31.31	45.25	31.9	25.1	42.86	30.53	39.21
PQQ28_RS10575	cheW	186.2	127.81	394.5	178.36	315.39	182.32	147.29	244.67	182.45	278.65
PQQ28_RS10600	cheY	104.36	69.4	131.12	94.08	138.7	70.46	72.94	117.03	76.2	126.22

KEGG enrichment identified that the treatment with 2 % RGD had activated several pathways of chemotaxis and metabolism pathways, which suggested chemotaxis and nutrient status are closely connected to each other (Fig. 1I–S2F, S2G). Gene set enrichment analysis (GSEA) indicated that the chemotaxis-associated bacterial (MAP02030), taxis (GO:0042330), and chemotaxis (GO:0006935) pathways were significantly enriched (Fig. 1J–L). There was agrees with bacteria observed within the 2 % RGD treatment being more mobile than others in this observation. There was an intermediate level of chemotactic activity on plain agar, which suggested that the nutrients served as signaling molecules [29]. These data suggest RGD treatment impacts the major chemotaxis-associated pathways, which is in agreement with the hypothesis that RGD plays a role in bacterial mobility and growth.

### 3.2 Characterization of the GelMA/CMCS-Ag/RGD hydrogel

**Fig. 2:**
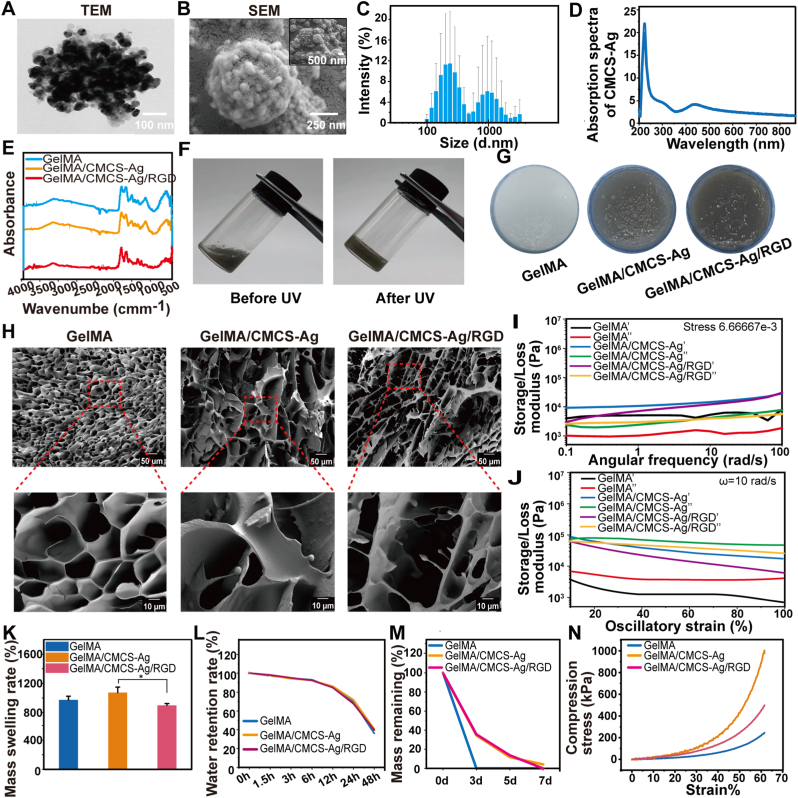
Characterization of the GelMA/CMCS-Ag/RGD hydrogel. (A) TEM image of CMCS-Ag (Scale = 100 nm). (B) SEM image of CMCS-Ag (Scale = 100 nm). (C) Particle size distribution of CMCS-Ag particles. (D) The absorbance spectrum of CMCS-Ag in the UV region. (E) Fourier transform infrared spectra of components of the GelMA/CMCS-Ag/RGD composite. (F) State transition of hydrogel during the crosslinking process under UV light. (G) Macroscopic photographs of GelMA, GelMA/CMCS-Ag, and GelMA/CMCS-Ag/RGD hydrogels. (H) SEM images of the GelMA, GelMA/CMCS-Ag, and GelMA/CMCS-Ag/RGD hydrogels (upper scale = 50 μm, lower scale = 10 μm). (I) Rheological behavior under various amplitudes (n = 3). (J) Rheological behavior under various frequencies (n = 3). (K) Swelling rate of composite hydrogels with different components (n = 3). (L) Water retention rates of composite hydrogels with different contents (n = 3). (M) Degradation characteristics of composite hydrogels with different contents (n = 3). (N) Compressive strengths of composite hydrogels with varying contents. (*∗P* < 0.05, *∗∗P* < 0.01, *∗∗∗P* < 0.001, ns: non-significant differences).

GelMA and CMCS are are typical natural polymeric materials with excellent biocompatibility [30]. We developed a chemotactic antibacterial wound dressing by incorporating silver nanocomposites into a GelMA and CMCS matrix, following a screening of chemotactic agents, to address wound infections. Silver ions were encapsulated into CMCS to form nanoparticles. TEM and SEM imaging showed that CMCS-Ag particles were spherical, with microspheres on their surface, and the particle size distribution of CMCS-Ag was uniform (Fig. 2A–C). A characteristic infrared absorption peak was observed at 420 nm, which is consistent with previous reports. (Fig. 2D), which was consistent with previous reports [31,32]. These results indicate that the synthesis of silver nanoparticles was successful. Then, we crosslinked GelMA/CMCS-Ag/RGD by UV light to form a hydrogel. The results showed that the GelMA/CMCS-Ag/RGD hydrogel possesses the darkest color (Fig. 2F–G). FTIR analysis revealed that the binding of RGD, CMCS-Ag, and GelMA was primarily achieved through physical bonds rather than chemical bonds (Fig. 2E). SEM image that CMCS-Ag particles had been embedded into the hydrogel (Fig. 2H).

Rheological testing showed that the storage modulus of all groups was higher than the loss modulus (Fig. 2I–J), indicating that they exhibited the characteristics of solid-type hydrogels. The introduction of CMCS-Ag can significantly improve the swelling properties (Fig. 2K and S3A) and compressive strength of the hydrogel (Fig. 2N and S3D-S3E). Additionally, the hydrogel exhibited excellent water retention properties (Fig. 2L and S3B), which are beneficial for maintaining a moist environment conducive to wound healing [33]. The degradation of the hydrogel was assessed under simulated physiological conditions (37 °C, in the presence of type I collagenase) [34]. The pure GelMA degraded completely within 3 d, while the GelMA/CMCS-Ag/RGD hydrogel took 7 d to degrade (Fig. 2M and S3C). Additionally, the GelMA/CMCS-Ag/RGD hydrogel maintained its structural integrity during degradation, which may attributable to the increased crosslinking density provided by CMCS-Ag. Clinically, a 7-day degradation cycle is suitable for acute wound healing, reducing the frequency of dressing changes, secondary trauma, and patient discomfort. For chronic or difficult-to-heal wounds, a longer degradation cycle may offer greater advantages. In summary, we successfully prepared a functional hydrogel, and characterized its chemotactic properties.

### 3.3 In vitro release properties of silver ions and RGD from hydrogels and their antibacterial performance

**Fig. 3:**
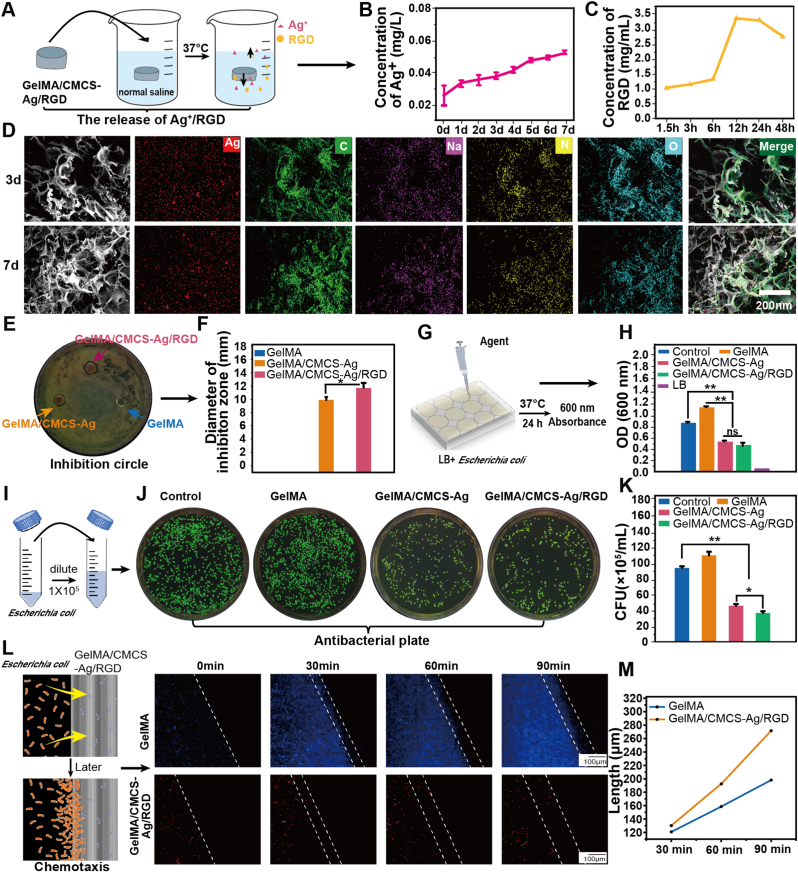
In vitro release properties of silver ions and RGD from hydrogels and their antibacterial performance. (A) Schematic diagram of Ag^+^ and RGD peptide release from hydrogels. (B) Ag^+^ release profile (n = 3). (C) RGD peptide release profile (At OD 562 nm). (D) Elemental analysis of hydrogels (3 d and 7 d). (E–F) Inhibition circle assay (16 h) and statistical analysis (n = 3). (G–H) Antimicrobial assessment (24 h) and OD_600_ values. (I–K) Dilution method, plating, and bacterial colony counting results (n = 3). (L–M) Fluorescence microscopy images (blue: *E. coli* labeled with blue fluorescence, red: *E. coli* labeled with red fluorescence) and quantification of bacterial migration distances. (*∗P* < 0.05, *∗∗P* < 0.01, *∗∗∗P* < 0.001, ns: non-significant differences). (For interpretation of the references to color in this figure legend, the reader is referred to the Web version of this article.)

Ag^+^ exhibits broad-spectrum antimicrobial activity, but excessively high local silver ion concentrations will be cytotoxicity [35]. To avoid this problem, we designed a hydrogel with bacterial chemotaxis and controlled Ag^+^ release. Silver ions were encapsulated into CMCS to form nanoparticles, which were then loaded on RGD peptides grafted GelMA. The bacteria in the wound site could be attracted to the hydrogel surface via RGD-mediated chemotaxis, followed by silver nanoparticle-mediated bacterial killing. First, the release of Ag^+^ and RGD were evaluated in a simulated saline at 37 °C (Fig. 3A). Over 7 days, Ag^+^ was continuously released, and its concentration was maintained below 0.06 mg/L, which is within the safe range (0.01–0.1 mg/L) (Fig. 3B). This may attribute to CMCS prevents the diffusion of high doses of Ag^+^, while photoreduction technology minimizes toxicity risks, thereby enhancing the biocompatibility of the hydrogel [36]. To further simulate in vivo enzymatic degradation, Ag+ and RGD release were also examined in a collagenase-containing environment (2 U/mL, 0.9 % NaCl). As shown in Fig. S4A–4C, both Ag^+^ and RGD exhibited slightly lower cumulative release compared to the enzyme-free condition. However, all reached the release peak at the third day. which might be due to the fact that most of the hydrogel began to degrade from the third day. Under simulated physiological conditions, the hydrogel completely degradation within approximately 7 days. Considering this degradation behavior and the typical 2-3-day clinical dressing replacement cycle, long-term Ag^+^ release monitoring beyond 7 days was not conducted. These results confirm that the hydrogel maintains sufficient antibacterial performance with minimal ion leakage, aligning with the concept of a “side-effect-free” antibacterial strategy.

The release of RGD reached a peak (∼3.25 mg/mL) at 12 h, then the release rate remained relatively stable (Fig. 3C and S3F). Consistent with the collagenase-containing test, RGD degradation slightly reduced cumulative release but did not compromise its functional persistence at the wound site. The sustained release of RGD peptides is particularly critical for wound healing, as it directly facilitates cell adhesion and migration at the wound site [27]. Elemental analysis of freeze-dried hydrogel samples after 3 and 7 days of immersion showed that the silver content slightly decreased from 1.11 wt% on day 3 to 0.83 wt% on day 7 (Fig. 3D and S4D), indicating controlled Ag^+^ release. In this study, Ag^+^ was chelated by the carboxyl and amino groups of CMCS, which effectively confined the ions within the hydrogel framework and prevented their excessive leakage. This mechanism contrasts with conventional antibacterial strategies that depend on the continuous release of silver ions. Once RGD induces bacterial chemotaxis toward the hydrogel surface, the bacteria are killed during direct contact with nanosilver. This ‘chemotaxis + contact-killing’ mode effectively reduces the likelihood of Ag^+^ entering the body fluid environment, thereby significantly lowering toxicity risk. In this study, the strategy “side-effect-free” specifically refers to the CMCS-Ag chelation design, which effectively prevents large amounts of silver ions into the body fluid environment. This strategy minimized potential harm to host cells and demonstrated both biosafety and efficacy in animal models.

Antibacterial efficacy was conducted by measuring the zone of inhibition and bacterial proliferation [37]. At 16 h, the hydrogel exhibited a small zone of inhibition, which disappeared by 24 h (Fig. 3E-F), indicating that the antibacterial efficacy of the hydrogel. The subsequent measurements of bacterial growth revealed a significant reduction in bacterial proliferation over time (Fig. 3G-K). The GelMA hydrogel acted as a nutrient source that promoted bacterial growth [38], whereas GelMA/CMCS-Ag/RGD hydrogel significantly enhanced antimicrobial efficacy. Notably, despite the nutrient properties of RGD, its addition did not weaken the antimicrobial function of the hydrogel. Instead, RGD enhanced *E. coli* aggregation, enabling silver ions to exert more efficient bactericidal activity. To further validate the antibacterial performance of the GelMA/CMCS-Ag/RGD hydrogel, in vitro inhibition-circle assays were conducted, including PA, SA and MRSA. As shown in Fig. S4E–4F, the hydrogel demonstrated contact-dependent bactericidal activity against all tested species, confirming its broad-spectrum antibacterial efficacy. In addition, the hydrogel was compared with Mepilex® antimicrobial soft silicone foam dressing (commercial product 1) and Atrauman® Ag dressing (commercial product 2). As illustrated in Fig. S4G–4H, the Mepilex® dressing exhibited negligible antibacterial activity, likely due to its porous and inert structure, whereas the Atrauman® Ag dressing displayed bactericidal efficiency comparable to that of our GelMA/CMCS-Ag/RGD hydrogel. These results indicate that our hydrogel achieves antibacterial performance equivalent to commercial silver dressings, while also providing unique advantages of chemotactic bacterial trapping and wound-healing promotion.

To visualize the chemotactic effect of on *E. coli*, we observed bacterial chemotaxis by using confocal fluorescence microscopy (Support movie). *E. coli* labeled with blue fluorescence was used as the control group (GelMA hydrogel), while *E. coli* labeled with red fluorescence was used for the experimental group (GelMA/CMCS-Ag/RGD hydrogel). Compared with the GelMA hydrogel, the GelMA/CMCS-Ag/RGD hydrogel exhibited a more pronounced chemotactic effect on *E. coli* (Fig. 3L-M). These findings indicated that the RGD peptide significantly enhances bacterial chemotaxis, thereby improving antimicrobial efficiency at the hydrogel interface.

### 3.4 Biocompatibility of the GelMA/CMCS-Ag/RGD hydrogel

**Fig. 4:**
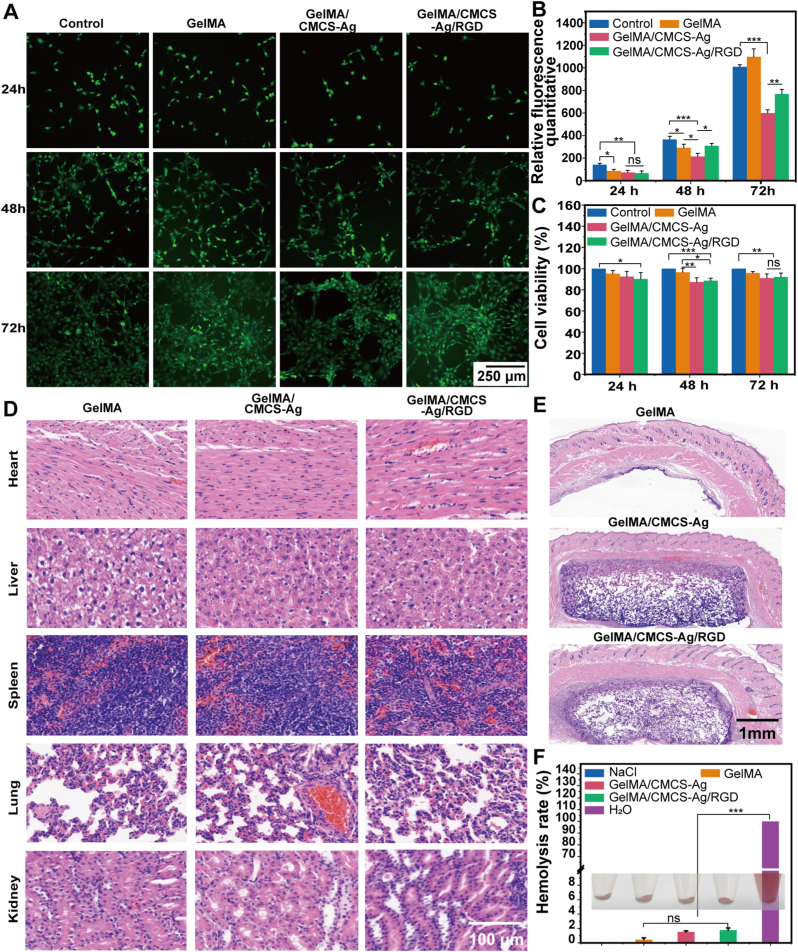
Biocompatibility of the GelMA/CMCS-Ag/RGD hydrogel. (A) Live/Dead staining images of NIH-3T3 cells cultured with different sets of hydrogels for 24, 48 and 72 h. The green color represents live cells. (B) Relative fluorescence quantitative analysis (n = 3) and (C) cell viability (n = 4) of different types of hydrogels co-cultured with NIH-3T3 for 1–3 d. Values represent mean ± standard deviation. (D) H&E staining of major organs of rats after the subcutaneous embedding experiment. (E) H&E staining of skin and subcutaneous tissues of rats in various groups of subcutaneous embedding experiment. (F) Hemolytic activity and hemolysis rate in culture with different groups of hydrogels (n = 3). (*∗P* < 0.05, *∗∗P* < 0.01, *∗∗∗P* < 0.001, ns: non-significant differences). (For interpretation of the references to color in this figure legend, the reader is referred to the Web version of this article.)

Biocompatibility is a fundamental property for clinical application [39]. Therefore, the biocompatibility of the GelMA/CMCS-Ag/RGD hydrogel was investigated. Prior studies indicate that HepG2 cells tolerate 12.5 ppm Ag^+^ concentrations, while cell viability drops sharply to around 7 % at 25 ppm [40]. The cytocompatibility of the GelMA/CMCS-Ag/RGD hydrogel extracts in NIH-3T3 cells were assessed by using live-dead cell staining and CCK-8 assays. The results indicated that the GelMA/CMCS-Ag/RGD hydrogel showed strong green fluorescence, indicating high cell viability and favorable cytocompatibility of the extracts (Fig. 4A-B). When the Ag^+^ concentration in the hydrogel extract was below 0.25 μg/mL (0.25 ppm), the survival rate of NIH-3T3 fibroblasts was well above 87 % (Fig. 4C). This indicated that the hydrogel possesses good biocompatibility.

Hydrogels were then implanted subcutaneously into rats to further evaluate their in vivo biocompatibility. H&E staining results at day 7 showed that the subcutaneously implanted hydrogels gradually degraded and no toxic side effects were founded (Fig. 4D-E). Hemocompatibility of the GelMA/CMCS-Ag/RGD hydrogel was evaluated using the hemolysis test [41]. The results showed that the hemolysis rate of the GelMA/CMCS-Ag/RGD hydrogel was only 3 % (Fig. 4F), which meets biomedical safety requirements. In summary, we have successfully developed a multifunctional hydrogel with good biocompatibility.

### 3.5 The effect of GelMA/CMCS-Ag/RGD on the healing of infectious skin wounds in mice

**Fig. 5:**
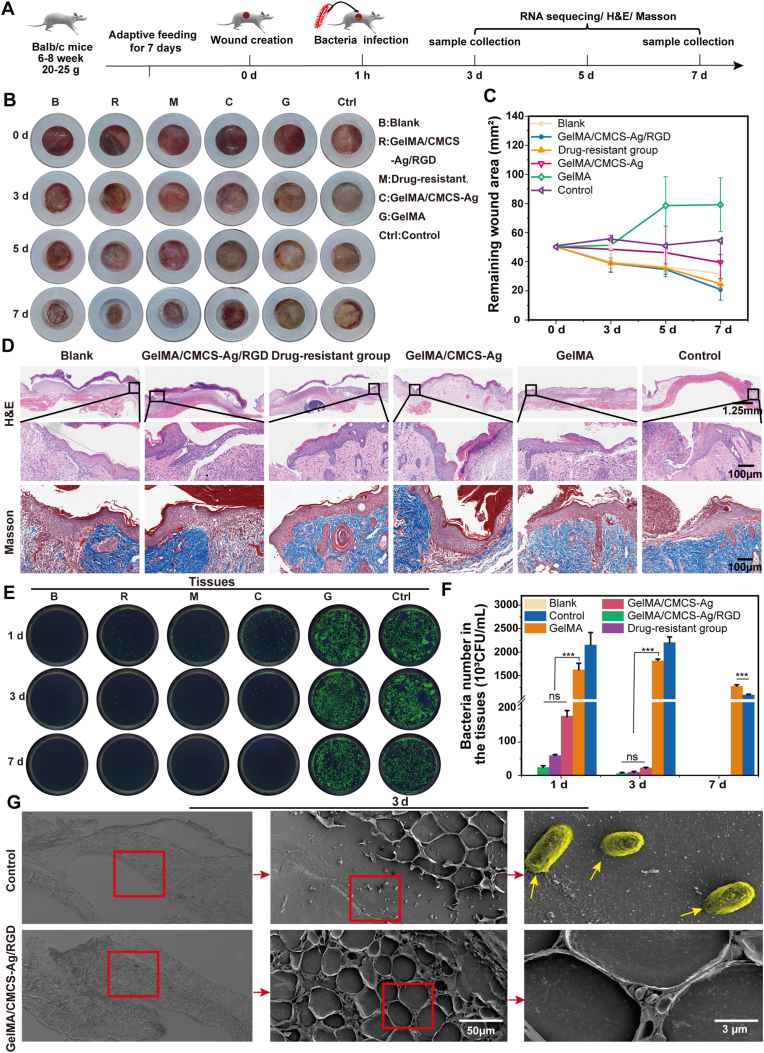
The effect of GelMA/CMCS-Ag/RGD on the healing of *E. coli*-infected skin wounds in mice. (A) Experimental schematic. (B) Representative images of wounds (blank group, GelMA/CMCS-Ag/RGD group, drug-resistant group, GelMA/CMCS-Ag group, GelMA group, control). (C) Statistical analysis of wound area (n = 3). (D) Representative H&E staining and Masson staining images of epithelium at days 3 and 7. (E–F) Images and quantitative analysis of bacteria from the tissue homogenates (n = 3). (G) Electron microscopy images of deep tissue on day 3. (*∗P* < 0.05, *∗∗P* < 0.01, *∗∗∗P* < 0.001, ns: non-significant differences).

We used a mouse full-thickness skin defect wound infection model to evaluate the therapeutic efficacy of the hydrogel (Fig. 5A). First, white concentric rings were used as an anti-contraction barrier to minimize the impact of mouse's skin contraction on wound healing [41]. Three days post-infection, the wounds in the control group and the GelMA group contained abundant purulent exudate, whereas those in the blank group and the GelMA/CMCS-Ag/RGD group contained only minimal purulent exudate and tended towards wound healing (Fig. 5B-C). This may be related to the water absorption capacity of the GelMA/CMCS-Ag/RGD hydrogel. By day 7, persistent pus was still observed in the control and GelMA groups, whereas the wounds in the other groups had begun to heal.

Since epithelialization is a critical step in wound healing [42], we analyzed epithelial regeneration on the wound at 3 and 7 days post-infection using H&E staining. The results showed that the length of newly formed epithelium in the GelMA/CMCS-Ag/RGD group and the resistant group was significantly higher than those in the control group and the GelMA group, and the same trend was observed at both time points (Fig. 5D and Fig. S5A). Additionally, the epithelial length in the antibiotic-resistant group was not significantly different from that in the blank group, while the epithelialization rate on the wound surface in the GelMA/CMCS-Ag/RGD group was faster, and the epithelial tissue length even exceeded that of the blank control group at both 3 and 7 days. Collagen deposition plays a key role in wound healing [43], as observed via Masson staining (Fig. 5D and S5B). The treatment group exhibited higher hair follicle and collagen fiber densities than the control group. These findings indicate enhanced myofibroblast activity and ordered collagen deposition during the early stages of proliferation.

Hydrogel's antimicrobial activity directly impacts wound healing rates [39]. We quantified bacterial loads on the wound surface and within tissue homogenates at days 1, 3, and 7 using standard plate counting methods (Fig. 5E-F and S5C-D). Compared with the control, GelMA, which likely providing nutrients, fostered bacterial proliferation on the wound surface, leading to significantly higher counts. In contrast, both the GelMA/CMCS-Ag/RGD group and the group infected with antibiotic-resistant bacteria showed significantly reduced bacterial counts on days 3 and 7. Strikingly, GelMA/CMCS-Ag/RGD achieved complete (100 %) bacterial clearance on the wound surface by day 3, demonstrating CMCS-Ag's potent bactericidal effect. Furthermore, even antibiotic-resistant *E. coli* was fully eradicated by day 7.

Analysis of internal tissue homogenates revealed higher bacterial loads deep within control group tissues compared to their own wound surfaces, indicating significant bacterial invasion. Conversely, in the GelMA/CMCS-Ag/RGD, antibiotic-resistant infection, and GelMA groups, the internal tissue counts were lower than surface counts. This pattern suggests that these hydrogels attract bacteria (chemotaxis) towards the material itself, trapping them near the surface and preventing deep tissue invasion. Critically, the internal counts in the GelMA/CMCS-Ag/RGD group were significantly lower than in the GelMA/CMCS-Ag group, highlighting RGD's specific role in boosting this chemotactic entrapment. SEM images of the tissues on days 3 and 7 (Fig. 5G and S5E) visually confirmed this phenomenon: the deep tissues in controls harbored numerous bacteria, whereas bacteria were absent from the deep tissues of all hydrogel-treated groups. The significant reduction of bacterial load in wound homogenates (Fig. 5E–F), together with the marked decrease of bacteria in deep tissues observed by transmission electron microscopy (Fig. 5G), indirectly supports the occurrence of the “programmatic trapping and killing” mechanism. These results suggest that the hydrogel can effectively adsorb and eliminate bacteria within infected tissues. Collectively, these findings demonstrate that the GelMA/CMCS-Ag/RGD hydrogel's dual mechanism - chemotactically attracting bacteria to the surface combined with potent bactericidal activity - effectively controls infection and promotes wound healing.

**Fig. 6:**
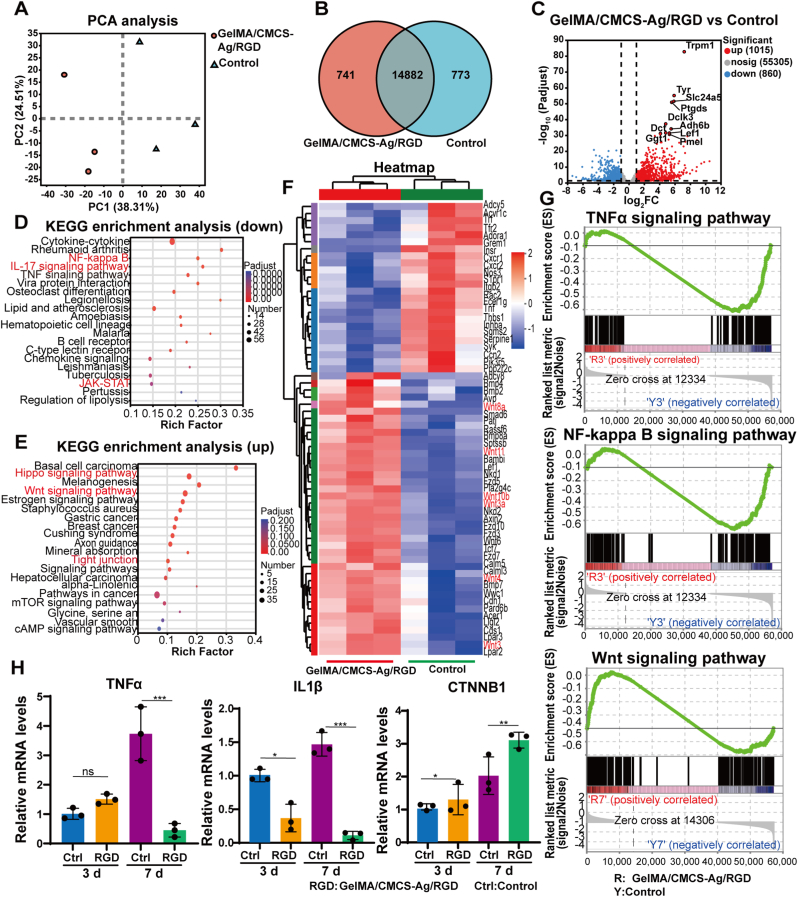
Mechanisms underlying GelMA/CMCS-Ag/RGD-mediated wound healing. (A) PCA of DEGs in treated vs. control wound tissues. (B) Venn diagram of overlapping DEGs. (C) Volcano plot of DEGs following GelMA/CMCS-Ag/RGD treatment. (D–E) KEGG pathway enrichment analysis of downregulated and upregulated DEGs. (F) Heatmap showing significant downregulation of inflammation and cell growth-related genes after GelMA/CMCS-Ag/RGD treatment. (G) GSEA plots for TNFα, NF-κB, and Wnt signaling pathways. (H) PCR validation analysis (RGD: GelMA/CMCS-Ag/RGD group; Ctrl: untreated control group) (n = 3). (*∗P* < 0.05, *∗∗P* < 0.01, *∗∗∗P* < 0.001, ns: non-significant differences).

To elucidate the molecular mechanisms by which the GelMA/CMCS-Ag/RGD hydrogel regulates wound healing, wound tissue samples were collected from the control group and hydrogel-treated group on days 3 and 7 for transcriptomic analysis. First, principal component analysis (PCA) was performed to confirm the reproducibility of the samples (Fig. 6A). Second, a Venn diagram revealed 14,882 overlapping differentially expressed genes (DEGs) between the control group and the GelMA/CMCS-Ag/RGD group (Fig. 6B). Volcano plot results further showed that, compared with the control group, the GelMA/CMCS-Ag/RGD group had 1015 upregulated genes and 860 downregulated genes on day 3 (Fig. 6C), while the difference in gene expression between the two groups was not significant on day 7 (Fig. S6A). KEGG enrichment analysis showed that downregulated genes in the GelMA/CMCS-Ag/RGD group were primarily associated with IL-17, NF-κB, JAK-STAT, and chemokine signaling pathways (Fig. 6D and S6B-S6D). However, upregulated genes were primarily enriched in the Hippo signaling and Wnt signaling pathways (Fig. 6E). The analysis of immune pathway enrichment may be related to the antibacterial effect of silver ions, while wound healing may be indirectly influenced by wound inflammation regulation mechanisms.

Heatmaps showed significantly higher expression of Wnt-related genes and lower expression of inflammation-related genes in the hydrogel-treated group (Fig. 6F). We observed enhanced activity of extracellular matrix (ECM)-related proteins following hydrogel treatment in protein interaction analyses (Fig. 6E). Because Wnt signaling critically regulates cell proliferation and angiogenesis [44], its activation likely improved nutrient supply, reduced inflammation, and accelerated wound healing [45]. Gene Set Enrichment Analysis (GSEA) indicated that GelMA/CMCS-Ag/RGD hydrogel strongly suppressed the IL-17, TNFα, and NF-κB signaling pathways, but activated Wnt signaling and cell cycle pathways (Fig. 6G and S6F-H). Quantitative PCR (qPCR) confirmed that TNFα and IL-1β expression dropped significantly in the GelMA/CMCS-Ag/RGD group relative to controls. Conversely, expression of CTNNB1, CCND1, FN1, and PKT2 increased (Fig. 6H and S7A). Beyond the marked suppression of inflammatory pathways, KEGG and GSEA enrichment further revealed significant activation of extracellular matrix (ECM) remodeling and angiogenesis-related signaling (VEGF, HIF-1), alongside Hippo pathways regulating proliferation and oxygen metabolism. These findings suggest that the GelMA/CMCS-Ag/RGD hydrogel accelerates wound repair through synergistic suppression of inflammation and activation of regenerative signaling networks. Together, these results demonstrate that the GelMA/CMCS-Ag/RGD hydrogel promotes wound healing via multiple mechanisms: Silver ions exert antibacterial effects to reduce infection; GelMA/CMCS maintains a moist regenerative microenvironment; and the composite further activates Wnt signaling pathways linked to cell proliferation, accelerating healing.

### 3.6 The effect of GelMA/CMCS-Ag/RGD hydrogel on infected rabbit skin wounds

**Fig. 7:**
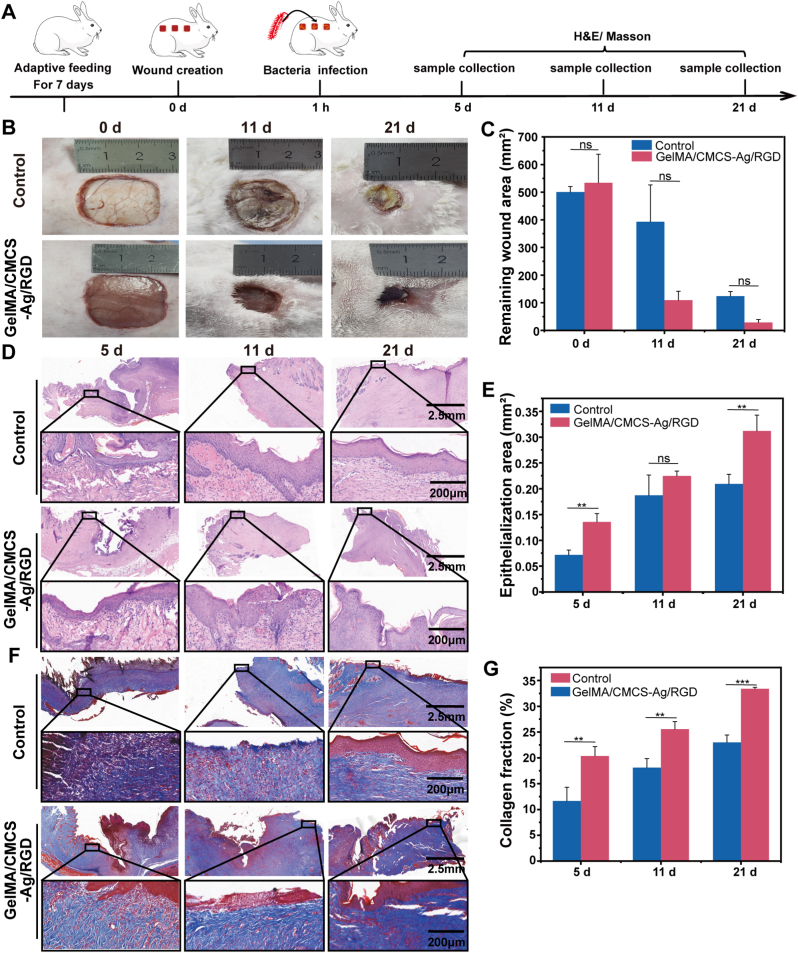
Assessment of wound healing in *E. coli*-infected rabbits. (A) Experimental schematic. (B) Representative images of wounds (GelMA/CMCS-Ag/RGD vs. control). (C) Quantitative wound area analysis (n = 3). (D) Representative H&E staining and (F) Masson staining images with statistical analysis of epithelialization (E) and collagen fraction (G) At 5, 11, and 21 d post-treatment (n = 3). (*∗P* < 0.05, *∗∗P* < 0.01, *∗∗∗P* < 0.001, ns: non-significant differences).

We further use *E. coli* infect rabbits full-thickness skin defects to test the hydrogel's antibacterial ability, as shown in Fig. 7A. In control group, wounds were infected after *E. coli* inoculation, while the experimental group (GelMA/CMCS-Ag/RGD hydrogel) wounds exhibited inhibited *E. coli* proliferation rates following hydrogel treatment. Statistical analysis of wound healing on days 11 and 21 revealed that the hydrogel-treated group healed faster than the control group (Fig. 7B and C). Further analysis of the treated wound tissue via H&E staining showed that the epithelialization rate and new epithelial area in the hydrogel-treated wounds were significantly increased compared to the control group on days 5 and 11, and the wounds in the hydrogel-treated group had been completely epithelialized by day 21 (Fig. 7D and E). Masson staining analysis revealed that collagen production in the hydrogel-treated group was significantly increased compared to the control group (*P* < 0.05). No obvious toxic side effects were observed in the main organs of the rabbits (Fig. S7B). The above results further confirm that the GelMA/CMCS-Ag/RGD hydrogel can effectively inhibit bacteria and promote wound healing without significant toxic side effects, demonstrating its potential for clinical application. While the use of n = 3 per group allowed for statistically significant results, it inevitably restricts the generalizability of the findings. This limitation should be considered, and larger-scale studies will be necessary to further validate these outcomes.

## 4 Conclusion

In this study, we describe a chemical antibacterial compound without side effects that can accurately capture and remove external bacteria and create an optimal healing microenvironment. The targeted aggregation of *E. coli* toward RGD peptides allows it to be effectively captured on the hydrogel surface, and Ag^+^ shows an effective antibacterial activity under temperature-controlled conditions. Ag^+^ in CMCS controllable release and significantly improves biocompatibility, showing an antibacterial application with "high-efficacy, low-toxicity" properties. The transcriptome validation confirms the induction of hydrogel-mediated regeneration pathways, including the remodeling of the extracellular matrix, vessel growth, and cell binding. In addition, the action of GelMA/CMCS Ag/RGD hydrogels was validated by rabbit models, which is important for its potential clinical transformation. While current work focuses on a monomicrobial *E. coli* infection, future research should address the problem of multibacterial wound pathogens. Overall, our results offer a biologically safe and innovative strategy for infection control and tissue regeneration, which offer a promising direction for the development of side-effect-free antibacterial biomaterials in clinical wound care.

## CRediT authorship contribution statement

**Zhiyi Liao:** Writing – original draft, Formal analysis, Data curation, Conceptualization. **Xinxin Su:** Software, Investigation, Formal analysis, Data curation. **Tingna Luo:** Formal analysis, Data curation. **Xiaohong Zhao:** Supervision, Methodology, Investigation. **Yicheng Guo:** Visualization, Validation, Software. **Xisheng Xu:** Resources, Project administration, Funding acquisition, Formal analysis. **Fan Wang:** Writing – review & editing. **Gaoxing Luo:** Writing – review & editing, Funding acquisition. **Rixing Zhan:** Writing – review & editing, Project administration, Funding acquisition.

## Data availability statement

The datasets generated and analyzed during the current study are available from the corresponding authors upon reasonable request.

## Ethics approval and consent to participate

All animal experiments were performed in accordance with the guidelines of the Ethics Committee of the First Affiliated Hospital of the Army Military Medical University, and were approved by the animal testing procedure (AMUWEC20224129).

## Funding

This work was supported by Chongqing Natural Science Foundation: CSTB2023NSCQ-MSX0458, State Key Laboratory of Trauma and Chemical Poisoning Foundation: SKLZZ202002, and Hunan Natural Science Foundation: 2025JJ70554.

## Declaration of competing interest

The authors declare no competing financial interests or personal relationships that could influence the work reported in this paper.

## Data Availability

Data will be made available on request.

## References

[1] Lv J.-C., Yang X., Zheng Z.-L., Wang Z.-G., Hong R., Liu Y., Luo E., Gou J.-X., Li L., Yuan B., Xu J.-Z., Li Z.-M. (2024). Engineering surface-adaptive metal–organic framework armor to promote infected wound healing. ACS Appl. Mater. Interfaces.

[2] J.C. Lv, Y. Qiu, D.M. Liang, H.C. Luo, Z.G. Wang, Y.W. Liu, J. Zhou, Q.J. Li, R. Hong, K. Li, J.Z. Xu, Z.M. Li, Unilateral surface-crystal-engineering induced dual-bionic janus multifunctional wound dressing for infected burn wound healing, Adv. Funct. Mater. (2025) Early View, doi: 10.1002/adfm.202503517.

[3] de la Fuente-Nunez C., Cesaro A., Hancock R.E.W. (2023). Antibiotic failure: beyond antimicrobial resistance. Drug Resist. Updates : reviews and commentaries in antimicrobial and anticancer chemotherapy.

[4] Chowdhury F.R., Findlay B.L. (2023). Fitness costs of antibiotic resistance impede the evolution of resistance to other antibiotics. ACS Infect. Dis..

[5] Buffie C.G., Bucci V., Stein R.R., McKenney P.T., Ling L., Gobourne A., No D., Liu H., Kinnebrew M., Viale A., Littmann E., van den Brink M.R., Jenq R.R., Taur Y., Sander C., Cross J.R., Toussaint N.C., Xavier J.B., Pamer E.G. (2015). Precision microbiome reconstitution restores bile acid mediated resistance to Clostridium difficile. Nature.

[6] Djebara S., Lavigne R., Pirnay J.P. (2025). Implementation challenges of personalised phage therapy. Lancet (London, England).

[7] Koncz M., Stirling T., Hadj Mehdi H., Méhi O., Eszenyi B., Asbóth A., Apjok G., Tóth Á., Orosz L., Vásárhelyi B.M., Ari E., Daruka L., Polgár T.F., Schneider G., Zalokh S.A., Számel M., Fekete G., Bohár B., Nagy Varga K., Visnyovszki Á., Székely E., Licker M.S., Izmendi O., Costache C., Gajic I., Lukovic B., Molnár S., Szőcs-Gazdi U.O., Bozai C., Indreas M., Kristóf K., Van der Henst C., Breine A., Pál C., Papp B., Kintses B. (2024). Genomic surveillance as a scalable framework for precision phage therapy against antibiotic-resistant pathogens. Cell.

[8] Laxminarayan R., Impalli I., Rangarajan R., Cohn J., Ramjeet K., Trainor B.W., Strathdee S., Sumpradit N., Berman D., Wertheim H., Outterson K., Srikantiah P., Theuretzbacher U. (2024). Expanding antibiotic, vaccine, and diagnostics development and access to tackle antimicrobial resistance. Lancet (London, England).

[9] Micoli F., Bagnoli F., Rappuoli R., Serruto D. (2021). The role of vaccines in combatting antimicrobial resistance. Nat. Rev. Microbiol..

[10] S.Y. Hu, J.C. Lv, M. Yan, J.Y. Wu, Z.G. Wang, R. Hong, J.X. Gou, L. Li, K. Li, J.Z. Xu, Z.M. Li, Multifunctional electrospun fiber sponge for hemostasis and infected wound healing, Small (2025) Early View, doi: 10.1002/smll.202409969. 10.1002/smll.202409969 40256833

[11] Ran B., Ran L., Wang Z., Liao J., Li D., Chen K., Cai W., Hou J., Peng X. (2023). Photocatalytic antimicrobials: principles, design strategies, and applications. Chem. Rev..

[12] Van den Bergh B., Michiels J.E., Wenseleers T., Windels E.M., Boer P.V., Kestemont D., De Meester L., Verstrepen K.J., Verstraeten N., Fauvart M., Michiels J. (2016). Frequency of antibiotic application drives rapid evolutionary adaptation of Escherichia coli persistence. Nat. Microbiol..

[13] Brouillard C., Bursztejn A.C., Latarche C., Cuny J.F., Truchetet F., Goullé J.P., Schmutz J.L. (2018). Silver absorption and toxicity evaluation of silver wound dressings in 40 patients with chronic wounds. J. Eur. Acad. Dermatol. Venereol. : JEADV.

[14] Keegstra J.M., Carrara F., Stocker R. (2022). The ecological roles of bacterial chemotaxis. Nat. Rev. Microbiol..

[15] Zhou B., Szymanski C.M., Baylink A. (2023). Bacterial chemotaxis in human diseases. Trends Microbiol..

[16] Xiao L., Guo Y., Wang F., Wang Y., Xu X., Ni W., Li B., Xing M., Luo G., Zhan R. (2021). A 3D chemotactic-thermo-promo bacterial hunting system: programmatic bacterial attract, capture, killing and healing the wound. Chem. Eng. J..

[17] Yu R., Zhang H., Guo B. (2021). Conductive biomaterials as bioactive wound dressing for wound healing and skin tissue engineering. Nano-Micro Lett..

[18] Wang K., Zhang Y., Chen T., Bai L., Li H., Tan H., Liu C., Qu X. (2023). Chain entanglement-driven tough, fatigue-resistant PEG-based injectable hydrogel adhesive for joint skin wound healing. Compos. B Eng..

[19] Norahan M.H., Pedroza-González S.C., Sánchez-Salazar M.G., Álvarez M.M., Trujillo de Santiago G. (2023). Structural and biological engineering of 3D hydrogels for wound healing. Bioact. Mater..

[20] Zhang X., Liang Y., Huang S., Guo B. (2024). Chitosan-based self-healing hydrogel dressing for wound healing. Adv. Colloid Interface Sci..

[21] Guo Y., Wang Y., Zhao X., Li X., Wang Q., Zhong W., Mequanint K., Zhan R., Xing M., Luo G. (2021). Snake extract-laden hemostatic bioadhesive gel cross-linked by visible light. Sci. Adv..

[22] He C., Yin M., Zhou H., Qin J., Wu S., Liu H., Yu X., Chen J., Zhang H., Zhang L., Wang Y. (2025). Magnetic nanoactuator-protein fiber coated hydrogel dressing for well-balanced skin wound healing and tissue regeneration. ACS Nano.

[23] Deng C., Zhang Q., He P., Zhou B., He K., Sun X., Lei G., Gong T., Zhang Z. (2021). Targeted apoptosis of macrophages and osteoclasts in arthritic joints is effective against advanced inflammatory arthritis. Nat. Commun..

[24] Qin W., Chandra J., Abourehab M.A.S., Gupta N., Chen Z.S., Kesharwani P., Cao H.L. (2023). New opportunities for RGD-engineered metal nanoparticles in cancer. Mol. Cancer.

[25] Duan L., Liu G., Liao F., Xie C., Shi J., Yang X., Zheng F., Reis R.L., Kundu S.C., Xiao B. (2025). Antheraea pernyi silk nanofibrils with inherent RGD motifs accelerate diabetic wound healing: a novel drug-free strategy to promote hemostasis, regulate immunity and improve re-epithelization. Biomaterials.

[26] Mesibov R., Adler J. (1972). Chemotaxis toward amino acids in *Escherichia coli*. J. Bacteriol..

[27] Ahmadi Z., Jha D., Yadav S., Singh A.P., Singh V.P., Gautam H.K., Sharma A.K., Kumar P. (2024). Self-assembled arginine-glycine-aspartic acid mimic peptide hydrogels as multifunctional biomaterials for wound healing. ACS applied materials & interfaces.

[28] Wang X., Vu A., Lee K., Dahlquist F.W. (2012). CheA–Receptor interaction sites in bacterial chemotaxis. J. Mol. Biol..

[29] Ni B., Colin R., Link H., Endres R.G., Sourjik V. (2020). Growth-rate dependent resource investment in bacterial motile behavior quantitatively follows potential benefit of chemotaxis. Proc. Natl. Acad. Sci. U. S. A..

[30] Li M., Liu R., Chen G., Wang H., Wang J., Kong B., Yu C. (2024). Mesenchymal stem cell exosome-integrated antibacterial hydrogels for nasal mucosal injury treatment. Research.

[31] Jin Y., Yang Y., Duan W., Qu X., Wu J. (2021). Synergistic and On-Demand release of Ag-AMPs loaded on porous silicon nanocarriers for antibacteria and wound healing. ACS applied materials & interfaces.

[32] Zhao L.J., Yu R.J., Ma W., Han H.X., Tian H., Qian R.C., Long Y.T. (2017). Sensitive detection of protein biomarkers using silver nanoparticles enhanced immunofluorescence assay. Theranostics.

[33] Liu Y., Yang X., Wu K., Feng J., Zhang X., Li A., Cheng C., Zhu Y.Z., Guo H., Wang X. (2025). Skin-inspired and self-regulated hydrophobic hydrogel for diabetic wound therapy. Adv. Mater..

[34] Srivastava N., Mohan R., Roy Choudhury A. (2025). A novel gellan-based nanoemulgel delivery system for co-encapsulation and in vitro digestion of hydrophilic/hydrophobic nutraceuticals. Carbohydrate polymers.

[35] Laomeephol C., Punjataewakupt A., Kanchanasin P., Phongsopitanun W., Ferreira H., Neves N.M., Aramwit P. (2025). Silver cross-linking of silk sericin-based hydrogels for improved stability and broad-spectrum antimicrobial properties. ACS Appl. Bio Mater..

[36] Cai Z., Song Y., Jin X., Wang C.C., Ji H., Liu W., Sun X. (2021). Highly efficient AgBr/h-MoO(3) with charge separation tuning for photocatalytic degradation of trimethoprim: mechanism insight and toxicity assessment. Sci. Total Environ..

[37] Haghniaz R., Rabbani A., Vajhadin F., Khan T., Kousar R., Khan A.R., Montazerian H., Iqbal J., Libanori A., Kim H.J., Wahid F. (2021). Anti-bacterial and wound healing-promoting effects of zinc ferrite nanoparticles. J. Nanobiotechnol..

[38] Shao L., Gao Q., Xie C., Fu J., Xiang M., He Y. (2020). Synchronous 3D bioprinting of large-scale cell-laden constructs with nutrient networks. Adv. Healthcare Mater..

[39] Li Y., Han Y., Li H., Niu X., Zhang D., Wang K. (2024). Antimicrobial hydrogels: potential materials for medical application. Small.

[40] Adeyemi J.A., Sorgi C.A., Machado A.R.T., Ogunjimi A.T., Gardinassi L.G.A., Nardini V., Faccioli L.H., Antunes L.M.G., Barbosa F. (2020). Phospholipids modifications in human hepatoma cell lines (HepG2) exposed to silver and iron oxide nanoparticles. Arch. Toxicol..

[41] Zhao X., Wu Z., Guo Y., Pu L., Pei Z., Liu Y., Hou B., Xie S., Luo G., Zhan R. (2025). Cell-laden biomimetic microneedles reconstruct skin rete ridge and stem cell niche. J. Nanobiotechnol..

[42] Peña O.A., Martin P. (2024). Cellular and molecular mechanisms of skin wound healing. Nat. Rev. Mol. Cell Biol..

[43] Li Q., Hu W., Huang Q., Yang J., Li B., Ma K., Wei Q., Wang Y., Su J., Sun M., Cui S., Yang R., Li H., Fu X., Zhang C. (2023). MiR146a-loaded engineered exosomes released from silk fibroin patch promote diabetic wound healing by targeting IRAK1. Signal Transduct. Targeted Ther..

[44] Zhang H., Nie X., Shi X., Zhao J., Chen Y., Yao Q., Sun C., Yang J. (2018). Regulatory mechanisms of the Wnt/β-Catenin pathway in diabetic cutaneous ulcers. Front. Pharmacol..

[45] Song L., Chang X., Hu L., Liu L., Wang G., Huang Y., Xu L., Jin B., Song J., Hu L., Zhang T., Wang Y., Xiao Y., Zhang F., Shi M., Liu L., Chen Q., Guo B., Zhou Y. (2023). Accelerating wound closure with metrnl in normal and diabetic mouse skin. Diabetes.

