# Supplementary material for: A side-effect-free chemotactic-antibacterial wound dressing for programmatic trapping, killing of bacteria, and wound repair
Source: Mater Today Bio. 2025 Nov 25;35:102594. doi: 10.1016/j.mtbio.2025.102594 (PMC12718208; doi:10.1016/j.mtbio.2025.102594)
Supplement: Multimedia component 1 [file mmc1.docx]

**A Side-Effect-Free Chemotactic-Antibacterial Wound Dressing for Programmatic Bacterial Trapping, Killing, and Wound Repair**

Zhiyi Liao^1, 2+^, Xinxin Su^3+^, Tingna Luo^1+^, Xiaohong Zhao^1^, Yicheng Guo^1^, Xisheng Xu^2^, Fan Wang^4^*, Gaoxing Luo^1^*, Rixing Zhan^1^*

^+^Zhiyi Liao, Xinxin Su, Tingna Luo these authors contributed equally to this work.

*Correspondence:

Rixing Zhan zhanrixing@tmmu.edu.cn, Gaoxing Luo logxw@tmmu.edu.cn, Fan Wang wangfan@tmmu.edu.cn.

^1^Institute of Burn Research, State Key Laboratory of Trauma and Chemical Poisoning, Southwest Hospital, The Third Military Medical University (Army Medical University), Chongqing 400038, China.

^2^Department of Burn and Plastic Surgery, the First People’s Hospital of Chenzhou, University of South China, Chenzhou 423000, China.

^3^Department of Burn, Plastic and Aesthetic Surgery,the first Affiliated Hospital of Guilin Medical University, Guilin, 541001 Guangxi, China

^4^Department of Plastic and Reconstructive Surgery, Southwest Hospital, The Third Military Medical University (Army Medical University), Chongqing 400038, China.

**This file includes:**

Supplementary Materials and Methods

Supplementary Figures S1 to S7

Support movie Control and Experiment group

**Supplementary** **Materials and Methods**

**Real-time fluorescence quantitative PCR**

EpiSCs and skin tissue samples were isolated through sequential phosphate-buffered saline (PBS, pH 7.4) rinses. Total RNA was extracted using the RNAprep Pure Cell/Bacteria Kit (TIANGEN Biotech Co., Ltd., Beijing) with subsequent spectrophotometric quantification (NanoDrop 1000, Thermo Fisher Scientific; A260/A280 ratio ≥1.8). Complementary DNA synthesis was performed via reverse transcription using PrimeScript RT Master Mix (Takara Bio, Dalian) followed by 1:10 dilution in nuclease-free water. Quantitative PCR amplifications were conducted in triplicate using TB Green Premix Ex Taq II (Takara Bio) on a CFX96 Real-Time system (Bio-Rad Laboratories) under standardized parameters: initial denaturation (95°C, 30 s); 40 cycles of 95°C (5 s)/60°C (30 s). Target genes included cell cycle regulators (p16INK4a, TP53, CDK4), oncogenic markers (KRAS, PRB, MYC), ECM markers (ITGβ1, Col1a1, LAMA4, FN1), FAK markers (PTK2), Wnt markers (Ctnnb1, Axin2), ERK markers (CCND1), and others (Notch1, TGFβ1, TNFα, IL1β), with NCBI accession numbers provided in Supplementary Table S1), normalized to glyceraldehyde-3-phosphate dehydrogenase (GAPDH). Relative quantification employed the 2−ΔΔCt method with inter-run calibrators and negative controls. Melt curve analyses confirmed amplification specificity, with threshold cycle values recorded when fluorescence exceeded baseline by 10 standard deviations.

**Table 1. Primers sequences used in RT- PCR.**

| **Gene** | **Primer sequences** |
| --- | --- |
| P16 | F: 5’-cgcgatgtcgcacggta-3’ |
|  | R: 5’-aatcggggatgtctgaggga-3’ |
| P53 | F: 5’-cctctccccagccaaagaag-3’ |
|  | R: 5’-tctcggaacatctcgaagcg-3’ |
| CDK4 | F: 5’-ttgtggccctcaagagtgtg-3’ |
|  | R: 5’-cagtcgcctcagtaaagcca-3’ |
| Kras | F: 5’-tcgacacagcaggtcaagag-3’ |
|  | R: 5’-caaagaaagccctccccagt-3’ |
| PRB | F: 5’-acatctcccaggagagtcca-3’ |
|  | R: 5’-ctgcttttgcattcgtgttcg-3’ |
| MyC | F: 5’-ccctccactcggaaggacta-3’ |
|  | R: 5’-gctggtgcattttcggttgt-3’ |
| ITGβ1 | R: 5’-gatccacaaaccgcaacct-3’ |
|  | F: 5’-tggtcagcaacgcatatctgg-3’ |
| Col1a1 | R: 5’-ccacgtctcaccattgggg-3’ |
|  | F: 5’-gctcctcttaggggccact-3’ |
| PTK2 | R: 5’-ctcgatctctcgatgagtgct-3’ |
|  | F: 5’-gagtacgtccctatggtgaagg-3’ |
| LAMA4 | R: 5’-aggattcgtactgttaccgtca-3’ |
|  | F: 5’-cagcgccaatgctacctgt-3’ |
| FN1 | R: 5’-gcccagtgagtttcagcaaagg-3’ |
|  | F: 5’-atgtggacccctcctgatagt-3’ |
| TGFβ1 | R: 5’-ctggcgagccttagtttggac-3’ |
|  | F: 5’-ccacctgcaagaccatcgac-3’ |
| Notch1 | R: 5’-tcgttgttgttgatgtcacagt-3’ |
|  | F: 5’-gatggcctcaatgggtacaag-3’ |
| Ctnnb1 | R: 5’-catctagcgtctcagggaaca-3’ |
|  | F: 5’-cccagtcttcacgcaagag-3’ |
| Axin2 | R: 5’-gagtgtaaagacttggtccacc-3’ |
|  | F: 5’-aacctatgcccggtttcctcta-3’ |
| CCND1 | R: 5’-acttgaagtaagatacggagggc-3’ |
|  | F: 5’-gcgtaccctgacaccaactc-3’ |
| TNFα | R: 5’-cgatcaccccgaagttcagtag-3’ |
|  | F: 5’-caggcggtgcctatgtctc-3’ |
| IL1β | R: 5’-tggatgctctcatcaggacag-3’ |
|  | F: 5’-gaaatgccaccttttgacagtg-3’ |
| GAPDH | F: 5’-cctggcacccagcacaat-3’ |
|  | R: 5’-gggccggactcgtcatac-3’ |

**Supplementary figure**

| 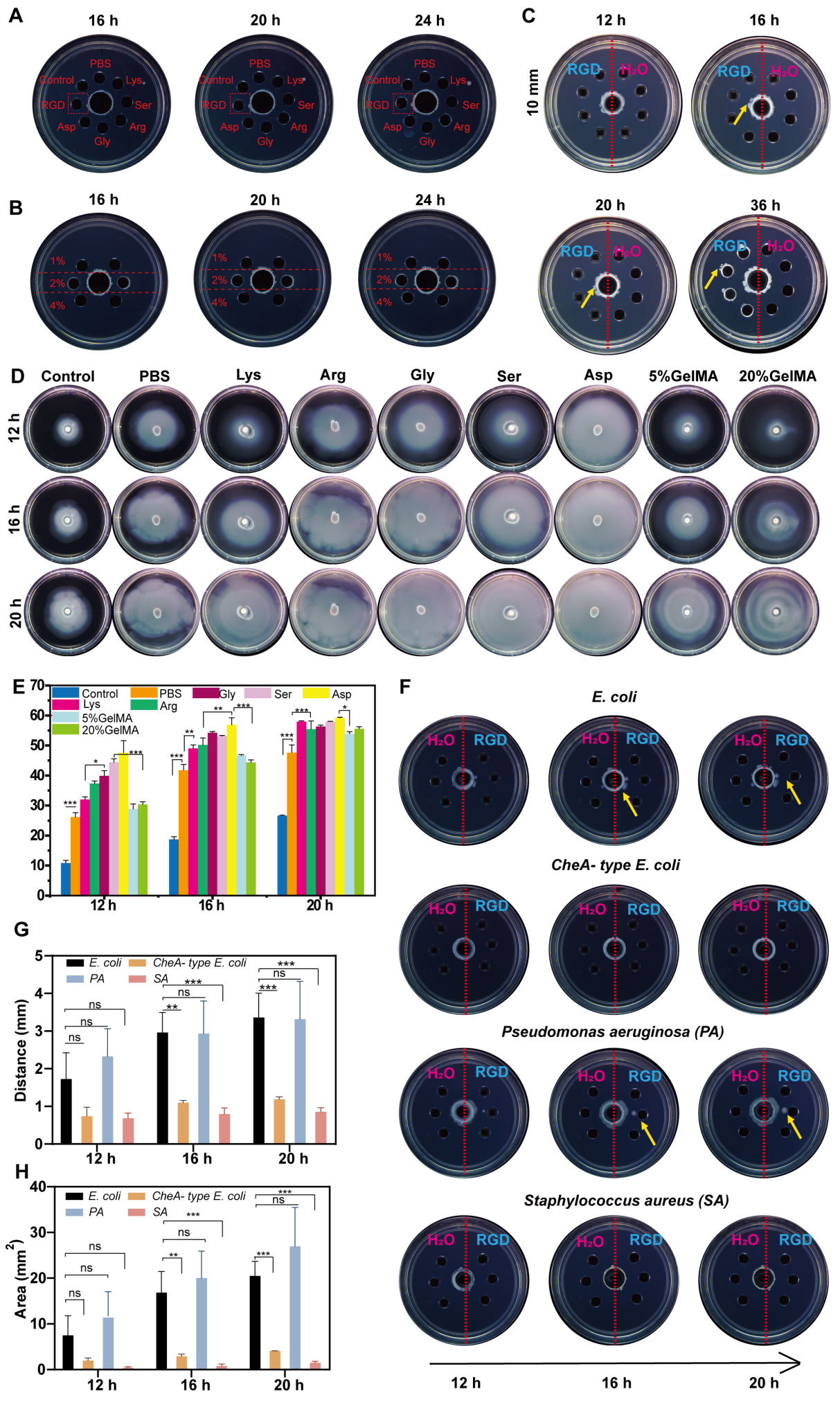 |
| --- |
| **Fig. S1. Supplementary evaluation of bacterial chemotaxis toward amino acids and RGD peptides.** (A) Chemotactic potential of Lys, Ser, Arg, Gly, Asp, PBS (n=3). (B) Chemotactic potential of RGD in 1%, 2%,4% (n=3). (C) Chemotactic potential of RGD peptides at 10 mm distances(n=3). Concentric circle experiments of each component in the chemotaxis plate (D) and related statistical graphs (E) (n=3). (F) Chemotactic effect of RGD peptide on *E. coli,* CheA^-^ type *E. coli, PA* and *SA* (n=3)*.* (G) Statistical result of chemotaxis distance (n=3). (H) Statistical result of chemotaxis area (n=3). (**P* < 0.05, ***P* < 0.01, ****P* < 0.001, ns: non-significant differences) |

| 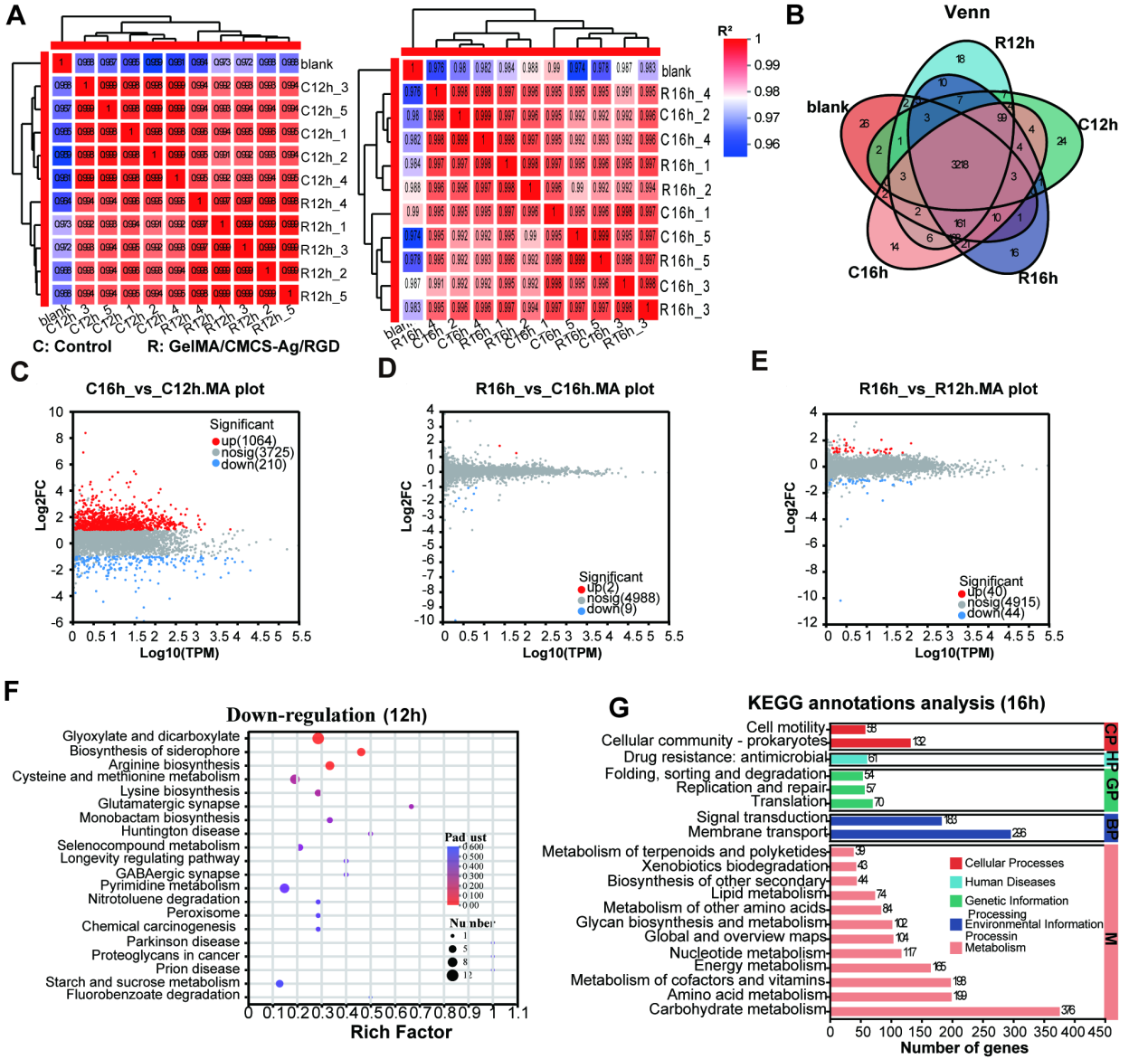 |
| --- |
| **Fig. S2. Transcriptomic analysis of chemotaxis-related gene expression in *E. coli*.** (A) Expression distribution of *E. coli* in each sample. (B) Venn diagram of differential gene. (C-E) Scatter plot of expression level differences. (F) KEGG enrichment analysis of *E. coli* in 12 h. (G) KEGG annotations analysis of *E. coli* in 16 h. |

| **Table 2.** Differential expression of core chemotaxis genes in E. coli. Normalized expression values (see Methods) for the canonical chemotaxis module-cheA (histidine kinase, PQQ28_RS10570), cheB (methylesterase, PQQ28_RS10595), cheW (adaptor, PQQ28_RS10575), and cheY (response regulator, PQQ28_RS10600)-are shown for five biological replicates per condition at 12 h and 16 h. Columns “R12h_1-5” and “R16h_1-5” denote the treatment group; “C12h_1-5” and “C16h_1-5” denote matched controls. |
| --- |
| **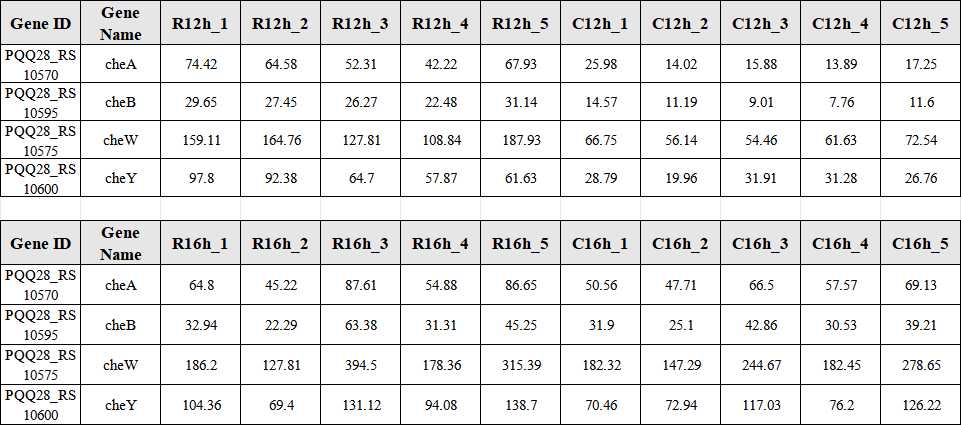** |

| **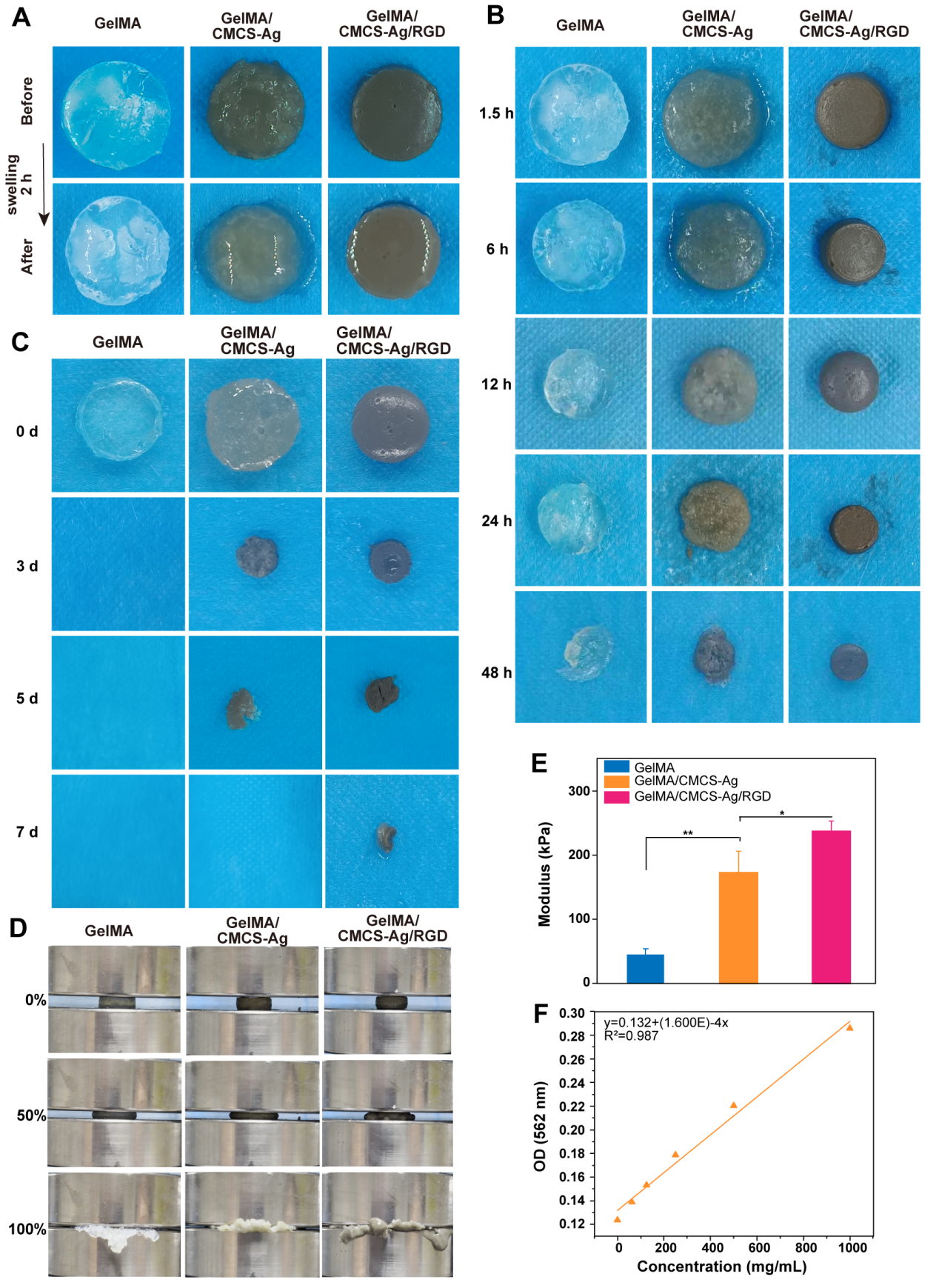** |
| --- |
| **Fig. S3. Supplementary characterization of hydrogel physicochemical and mechanical properties.** (A) Hydrogel swelling test procedure. (B) Hydrogel water retention test. (C) Testing of hydrogel degradation properties in the presence of a line of collagenases. (D) Universal tensile testing machine to test the compression process of hydrogel. (E) Hydrogel compression modulus (n=3). (F) Concentration calibration curve of RGD at OD562 nm. (P < 0.05 (*), P < 0.01 (**), P < 0.001 (***), ns: non-significant differences) |

| **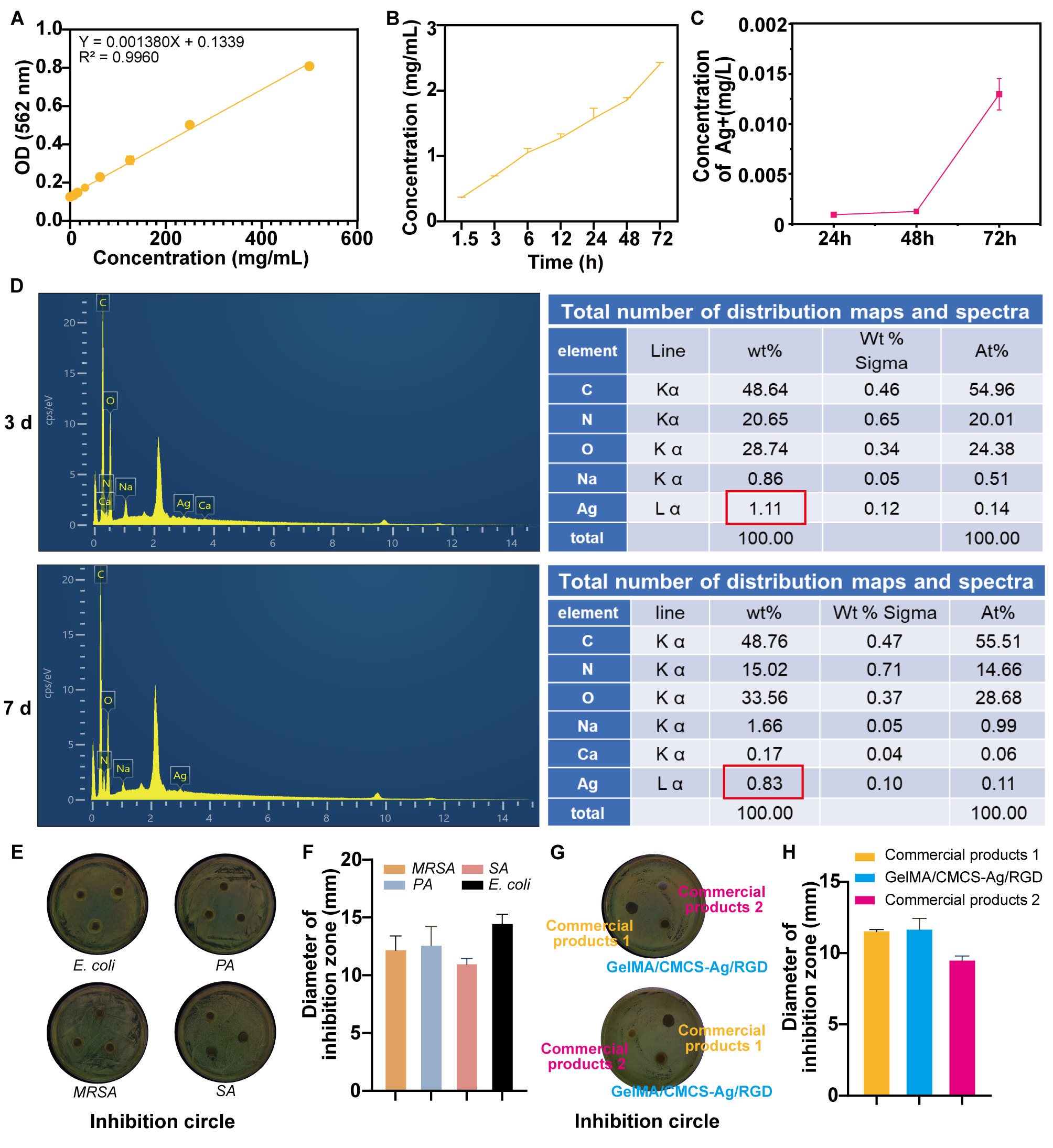** |
| --- |
| **Fig. S4. Elemental composition analysis of hydrogel cross-sections at different time points.** (A) Concentration calibration curve of RGD at OD 562 nm. (B) RGD peptide release profile (At OD 562 nm, Type I collagenase) (n=3). (C) Ag^+^ release profile ( Type I collagenase) (n=3). (D) Statistical comparison of elemental content in the hydrogel cross-section after immersion for 3 days and 7 days, respectively. (E) The inhibition circle assay of hydrogel against four types of bacteria and statistical analysis (F) (n=3). (G) Comparison of antibacterial effects between GelMA/CMCS-Ag/RGD and two commercial dressings (n=3). (P < 0.05 (*), P < 0.01 (**), P < 0.001 (***), ns: non-significant differences) |

| **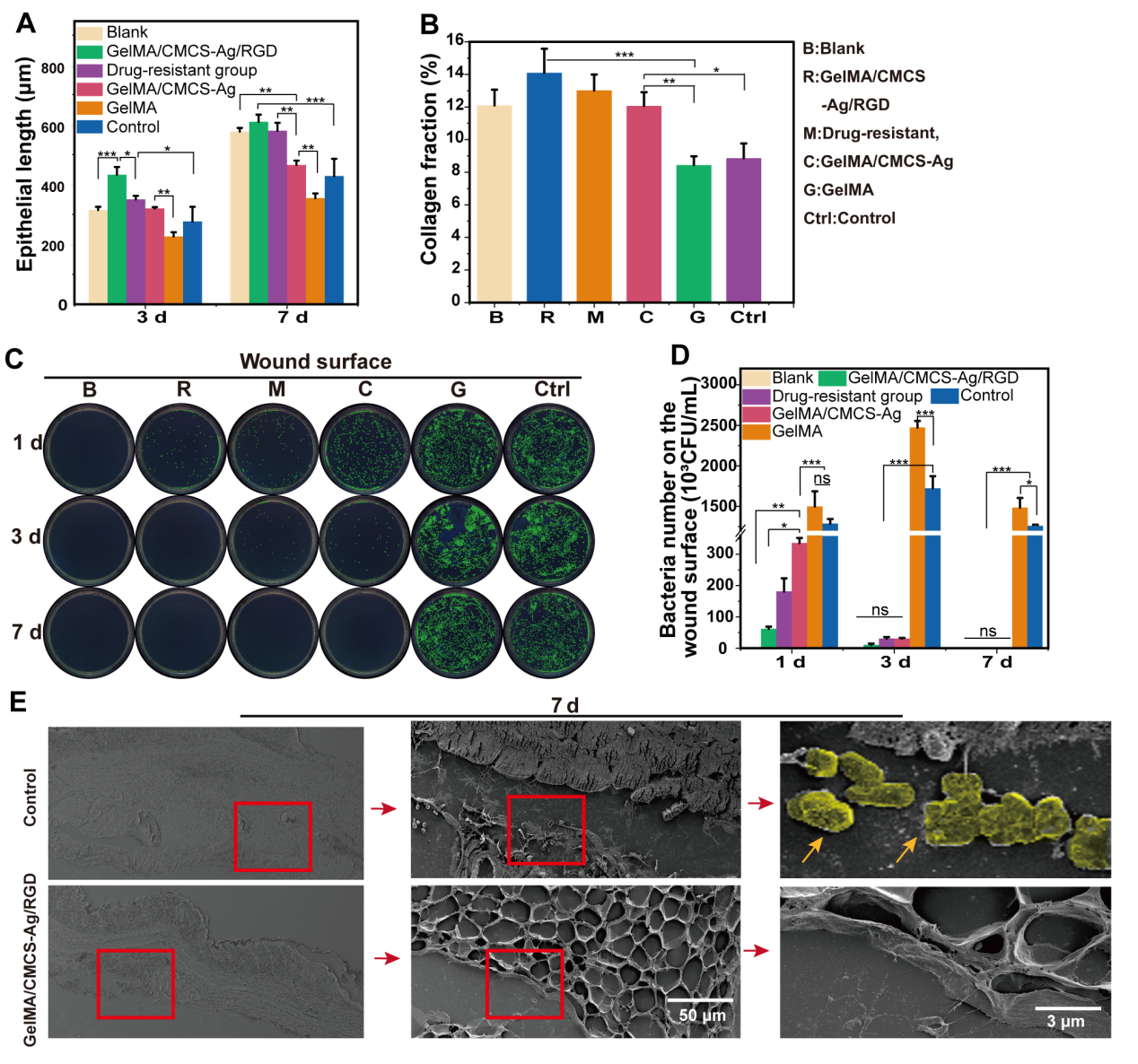** |
| --- |
| **Fig. S5. Supplementary evaluation of wound healing and bacterial distribution in *E. coli*-infected mouse wounds.** (A) Statistics on the length of neoplastic epithelium in mouse trauma (n=3). (B) Quantification of collagen deposition in the wound areas of each treatment group (n=3). (C–D) Representative images and statistical analysis of bacterial distribution on the wound surface (n=3). (E) Electron microscopic scanning of deep tissue in the trauma of mice on the 7 d. (P < 0.05 (*), P < 0.01 (**), P < 0.001 (***), ns: non-significant differences) |

| **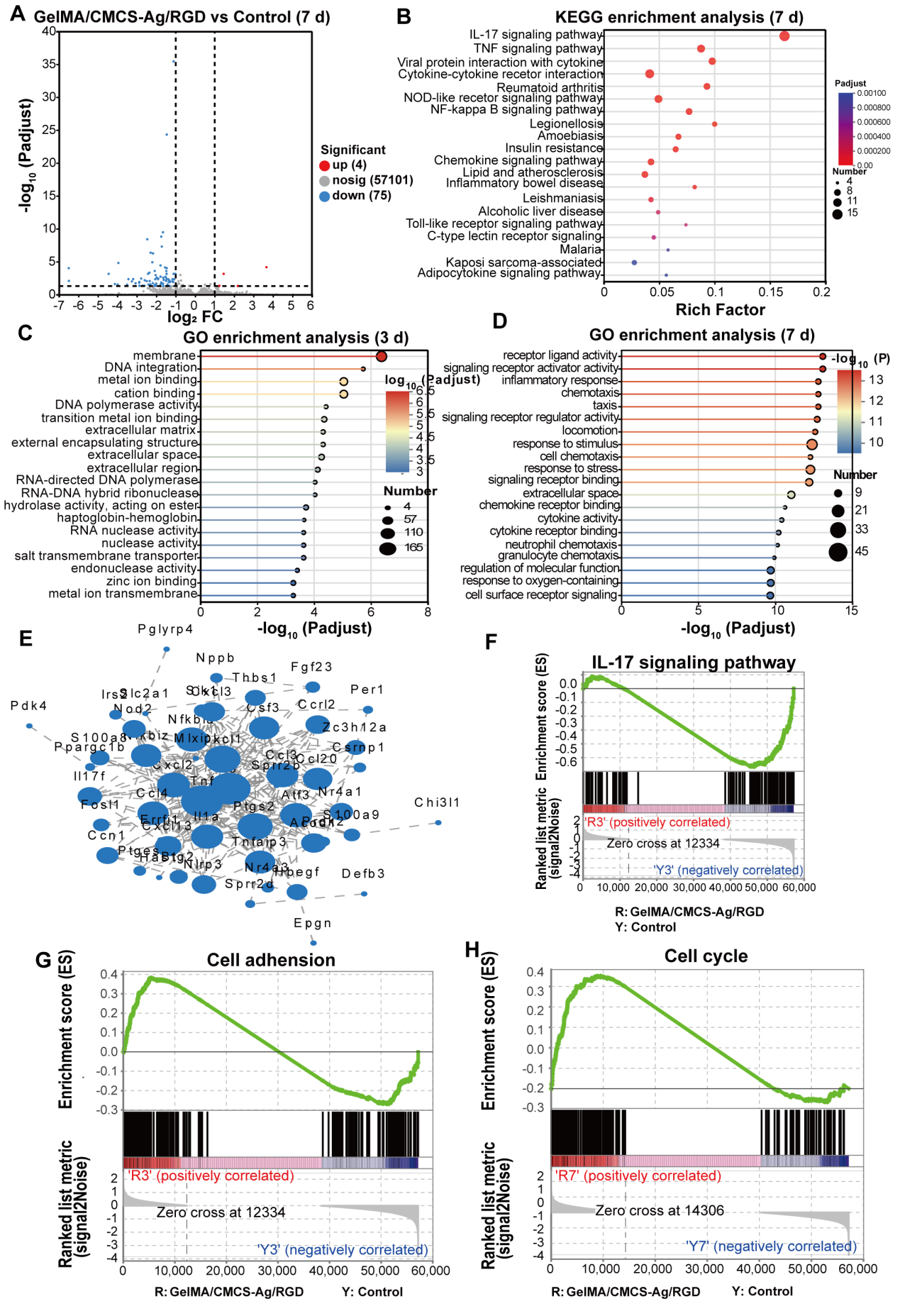** |
| --- |
| **Fig. S6. Transcriptomic and pathway analysis of mouse wound tissues treated with GelMA/CMCS-Ag/RGD hydrogel.** (A) Volcano plots were generated to show up and down-regulated genes after GelMA/CMCS-Ag/RGD treatment (7d). (B) KEGG pathway enrichment analysis of DEGs (7d). (C) GO pathway enrichment analysis of DEGs (3d). (D) GO pathway enrichment analysis of DEGs (7d). (E) Protein interaction network analysis (PPI) on differentially expressed genes in mouse wounds treated with GelMA/CMCS-Ag/RGD or control. (F-E) GSEA plot showing the genes of mice are significantly enriched in pathways that inhibit inflammation and promote proliferation after GelMA/CMCS-Ag/RGD treatment. |
| 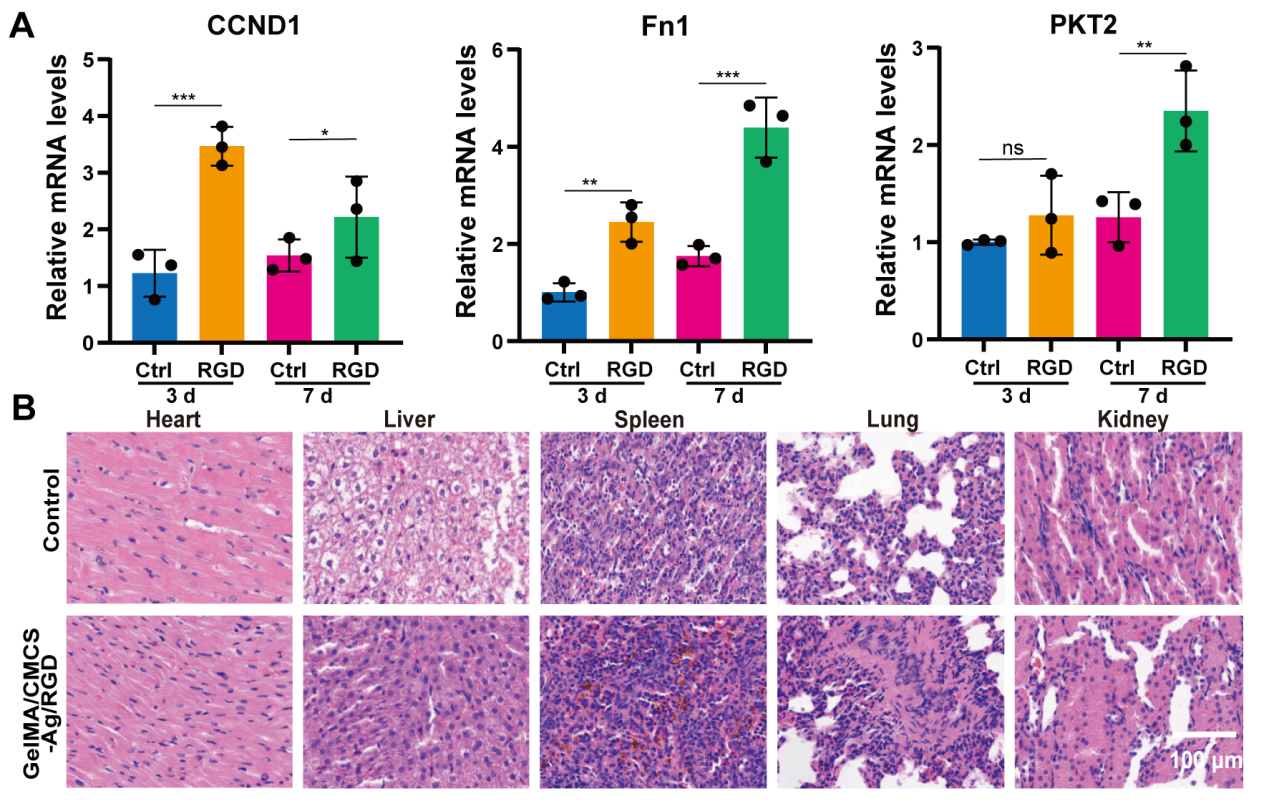 |
| **Fig. S7. Biosafety verification of GelMA/CMCS-Ag/RGD hydrogel.** (A) PCR analysis for verification. RGD means group of mice after GelMA/CMCS-Ag/RGD treatment, correspondingly, Ctrl means the control group of micewithout any treatment (n=3). (B) H&E staining of major organs of rabbits after subcutaneous embedding experiment. (P < 0.05 (*), P < 0.01 (**), P < 0.001 (***), ns: non-significant differences) |
